# Antiviral RNA interference in disease vector (Asian longhorned) ticks

**DOI:** 10.1371/journal.ppat.1010119

**Published:** 2021-12-03

**Authors:** Yan Xu, Zhengwei Zhong, Yanxin Ren, Liting Ma, Zhi Ye, Chuang Gao, Jingwen Wang, Yang Li

**Affiliations:** 1 CAS Key Laboratory of Animal Ecology and Conservation Biology, Institute of Zoology, Chinese Academy of Sciences, Beijing, China; 2 State Key Laboratory of Genetic Engineering, School of Life Sciences, Fudan University, Shanghai, China; Radboud University Medical Center, NETHERLANDS

## Abstract

Disease vectors such as mosquitoes and ticks play a major role in the emergence and re-emergence of human and animal viral pathogens. Compared to mosquitoes, however, much less is known about the antiviral responses of ticks. Here we showed that Asian longhorned ticks (*Haemaphysalis longicornis*) produced predominantly 22-nucleotide virus-derived siRNAs (vsiRNAs) in response to severe fever with thrombocytopenia syndrome virus (SFTSV, an emerging tick-borne virus), Nodamura virus (NoV), or Sindbis virus (SINV) acquired by blood feeding. Notably, experimental acquisition of NoV and SINV by intrathoracic injection also initiated viral replication and triggered the production of vsiRNAs in *H*. *longicornis*. We demonstrated that a mutant NoV deficient in expressing its viral suppressor of RNAi (VSR) replicated to significantly lower levels than wildtype NoV in *H*. *longicornis*, but accumulated to higher levels after knockdown of the tick Dicer2-like protein identified by phylogeny comparison. Moreover, the expression of a panel of known animal VSRs *in cis* from the genome of SINV drastically enhanced the accumulation of the recombinant viruses. This study establishes a novel model for virus-vector-mouse experiments with longhorned ticks and provides the first *in vivo* evidence for an antiviral function of the RNAi response in ticks. Interestingly, comparing the accumulation levels of SINV recombinants expressing green fluorescent protein or SFTSV proteins identified the viral non-structural protein as a putative VSR. Elucidating the function of ticks’ antiviral RNAi pathway *in vivo* is critical to understand the virus-host interaction and the control of tick-borne viral pathogens.

## Author summary

Tick-borne diseases (TBDs) are the most common illnesses transmitted by ticks, and the annual number of reported TBD cases continues to increase. The Asian longhorned tick, a vector associated with at least 30 human pathogens, is native to eastern Asia and recently reached the USA as an emerging disease threat. Newly identified tick-transmitted pathogens continue to be reported, raising concerns about how TBDs occur. Interestingly, tick can harbor pathogens without being affected themselves. For viral infections, ticks have their own immune systems that protect them from infection. Meanwhile, tick-borne viruses have evolved to avoid these defenses as they establish themselves within the vector. Here, we show in detail that infecting longhorned ticks with distinct arthropod-borne RNA viruses through two approaches natural blood feeding and injection, all induce the production of vsiRNAs. Dicer2-like homolog plays a role in regulating antiviral RNAi responses as knocking down of this gene enhanced viral replication. Furthermore, we demonstrate that tick antiviral RNAi responses are inhibited through expression heterologous VSR proteins in recombinant SINV. We identify both the virus and tick factors are critical components to understanding TBDs. Importantly, our study introduces a novel, *in vivo* virus-vector-mouse model system for exploring TBDs in the future.

## Introduction

Ticks are notoriously hematophagous ectoparasites, transmitting a broader spectrum of infectious agents to humans and animals [1–4]. Asian longhorned ticks is a three-host tick, with a wide distribution in ten countries, predominantly in eastern Asia, the USA, Australia, and New Zealand [5]. At least 30 human pathogens are associated with *H longicornis*, including six species of virus, such as thogoto virus (*Orthomyxoviridae*) [6], lymphocytic choriomeningitis virus (*Arenaviridae*) [7], nairobi sheep disease virus (*Nairoviridae*) [8], tick-borne encephalitis virus (*Flaviviridae*) [9] and particularly SFTSV (*Phenuiviridae*), which is closely related to Heartland virus in the USA [5,10]. Severe fever with thrombocytopenia syndrome (SFTS) is an emerging fatal disease with severe clinical symptoms including dyspnea, hemorrhagic or neurological signs [11–13]. After SFTS first emerged in China [11], confirmed cases were subsequently reported in South Korea [14], Japan [15] and recently in Vietnam [16], which raise concerns about this disease becoming global pandemics. Greater than 8000 clinical cases were reported in China by the end of 2018, and greater than1000 clinical cases had been reported in other Asian countries to date with high mortality rates ranging from 5% up to 30% [17]. Unfortunately, no efficient vaccines and antiviral drugs are available hitherto [13,18]. Considering the profound impact on public health, the World Health Organization has listed SFTS as one of the top ten viral diseases that require urgent and in-depth research [19].

The etiological agent of SFTS is SFTSV, which is a member of the Huaiyangshan Banyangvirus species, *Banyangvirus* Genus, *Phenuiviridae* family [20]. The genome of SFTSV contains tripartite negative-sense or ambisense RNA segments, including a large (L) segment encoding RNA-dependent RNA polymerase (RdRp), a medium (M) segment encoding glycoprotein precursor and a small (S) segment encoding nonstructural protein (NS) and nucleoprotein (NP) [21,22]. Existing evidence suggests that *H*. *longicornis* is the competent vector and plays a crucial role in the transmission of SFTSV [23–25]. Recently, this tick species has been found in 12 states of United States, which raises the public health concern about potential SFTSV transmission in North America [5]. Due to a shortage of tick genome sequence resources, to date, little is known about the molecular mechanisms involved with antiviral response in *H*. *longicornis*. Although increasing attention has been paid to the pathogenesis of SFTSV in mammals, the interaction between SFTSV and its arthropod vector remains to be investigated [5,26]. When we were preparing this manuscript, six high-quality ixodid tick genomes, including the *H*. *longicornis*, were open to the public [3]. With these tick genome resources, it may be feasible to identify more immune response relative genes to better understand the tick antiviral pathway.

Three primary innate immunity signaling pathways, Janus kinase/signal transducer and activator of transcription (JAK/STAT), Toll, and Immune deficiency (Imd) have been demonstrated antiviral defenses in arthropods [27–33]. While RNAi is considered to be the most important antiviral immune response in *Drosophila melanogaster* [34–39] and mosquitoes [40–44]. Dicer-2 is responsible for the sensing of virus-derived double-stranded RNA (dsRNA) and processing them into a pool of small interfering RNA (siRNA) duplexes. Then Ago-2, the core protein of the RNA-induced silencing complex (RISC), binds to the guide strand of a siRNA and mediates the endonucleolytic cleavage of complementary viral RNA to execute antiviral defense [45]. As a counterdefensive strategy, viruses have evolved VSRs, which antagonize antiviral RNAi through diverse ways [36,38,46–50]. Notably, bioinformatic analysis suggested that putative proteins involved in RNAi also exist in ticks’ genome [1,51,52]. Initial two studies investigating antiviral RNAi in *I*. *scapularis* tick uncovered abundant vsiRNAs with a peak length of 22 nt upon flavivirus infections [52,53]. Whether other families of arthropod-borne viruses can also induce vsiRNAs in ticks is not known.

Given the well-characterized genetic backgrounds and mature reverse genetic operating systems, model viruses have been extensively used in exploring the virus-host interaction, including antiviral RNAi. For instance, SINV, a prototypical *alphavirus* of the family *Togaviridae* with a non-segmented positive sense RNA genome [54] has been successfully utilized to investigate antiviral RNAi in *D*. *melanogaster* [35,55,56] and mosquitoes [40–42,57]. NoV is a mosquito-borne positive-sense RNA virus with two genomic RNA segments: RNA1 and RNA2. RNA1 encodes RdRp, which is responsible for viral genome replication and the synthesis of subgenomic RNA3, which subsequently produces the crucial non-structural viral protein B2, a potent VSR [58–60]. NoV is a unique member of the family *Nodaviridae* with the ability to cause lethal infection both in insects and suckling mice, and is also widely used in antiviral RNAi research [60–62]. Although the antiviral responses can be virus-vector dependent and tick is not a natural host for model viruses, considering their excellent performance in invertebrates and vertebrates, it may be feasible to dissect antiviral RNAi in ticks using these model viruses.

Here, we provide evidence that RNAi functions as an antiviral defense in *H*. *longicornis* ticks *in vivo* and NoV B2 protein acts as a suppressor to inhibit the production of vsiRNAs. The putative Dicer2-like protein in *H*. *longicornis* is involved in the tick antiviral RNAi. Significantly, we demonstrate that SFTSV infection stimulates the RNAi immune response through natural blood feeding, and the NS protein may be a VSR. Due to the lack of efficient vaccines and antiviral therapies in most tick-borne arboviruses diseases, understanding how arboviruses interact with arthropod vectors may provide feasible intervention measures for vector control.

## Results

### Longhorned ticks produce a distinct population of 22-nt viral siRNAs to target RNA viruses acquired through blood feeding

**Fig 1:**
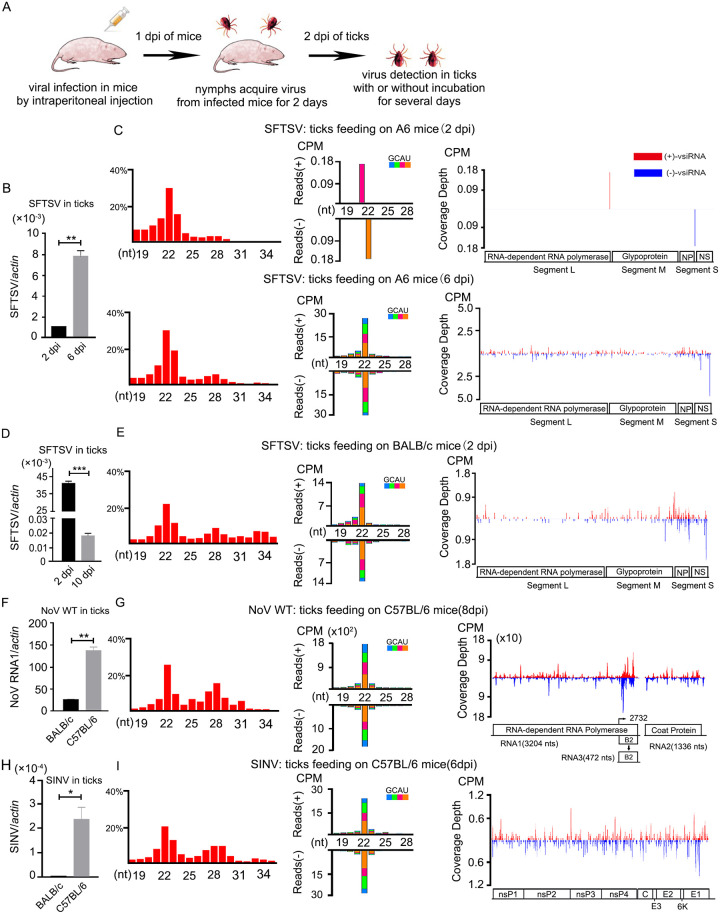
Profiles of vsiRNAs in infected *H*. *longicornis* ticks through blood feeding. (A) Schematic overview of viral acquisition by ticks through blood feeding. Briefly, nymphs were infected with the virus by blood feeding for two days on the virus-infected suckling mice. For infected ticks, the infection time was marked as 2dpi when ticks were removed from mice. If removed ticks were incubated for several days, the infection time was marked as (2+incubation days) dpi. (B) Viral RNA levels of SFTSV in ticks at 2dpi and 6dpi (incubation for 4 days) after sucking on infected A6 mice were determined by RT-qPCR. (C) Size distribution of total reads (left), virus-derived small RNAs (middle) and genomic coverage depth of 21- to 23-nt vsiRNAs (right) sequenced from ticks infected with SFTSV by blood feeding at 2dpi and 6dpi. For negative strand RNA virus, SFTSV, (+)-vsiRNA means antigenome vsiRNA. (D) Viral RNA levels of SFTSV in ticks at 2dpi and 10dpi (incubation for 8 days) after sucking on infected BALB/c mice were determined by RT-qPCR. (E) Size distribution of total reads (left), virus-derived small RNAs (middle) and genomic coverage depth of 21- to 23-nt vsiRNAs (right) sequenced from ticks infected with SFTSV by blood feeding at 2dpi. (F) Viral RNA levels of NoV WT in ticks at 8dpi (incubation for 6 days) after sucking on infected Balb/c or C57BL/6 mice were determined by RT-qPCR. (G) Size distribution of total reads (left), virus-derived small RNAs (middle) and genomic coverage depth of 21- to 23-nt vsiRNAs (right) sequenced from ticks at 8dpi infected with NoV WT by feeding on C57BL/6 mice. (H) Viral RNA levels of SINV in ticks at 6dpi (incubation for 4 days) after sucking on infected Balb/c or C57BL/6 mice were determined by RT-qPCR. (I) Size distribution of total reads (left), virus-derived small RNAs (middle) and genomic coverage depth of 21- to 23-nt vsiRNAs (right) sequenced from ticks at 6dpi infected with SINV by feeding on C57BL/6 mice. For RT-qPCR analyses, the viral RNA levels were calculated by ΔCt method and tick *β-actin* mRNA as the internal reference. The data represent mean±SEM values of three biological replicates (10 nymphs per biological replicate). Significance was determined by the Mann-Whitney test in (B) (D) (F) and (H). *P<0.05, ** P<0.01, *** P<0.001. For small RNA libraries, read counts are shown as per million total 18- to 28-nt reads (CPM), and the 5’ terminal nucleotide of virus-derived small RNAs is indicated by different colors. Genomic coverage depth is indicated by the position of its 5’ terminal nucleotide. Sense strand-vsiRNAs are depicted in red, and antisense strand-vsiRNAs are depicted in blue. A schematic diagram representing the organization of SFTSV, NoV, and SINV are presented.

*H*. *longicornis* mainly acquire and transmit SFTSV through blood feeding [23]. However, the interactions between SFTSV and *H*. *longicornis* are not completely understood, especially in the antiviral RNAi pathway. We therefore exploited a tick-mouse acquisition model to investigate whether SFTSV infection induce antiviral RNAi in a *bona fide* natural infection route (Fig 1A). Ticks were allowed to acquire virus by feeding on SFTSV burdened A6 (type I interferon receptor knockout C57BL/6) mice [63] (S1A Fig). Compared to viral replication levels at 2 days post incubation (dpi), SFTSV accumulated remarkably in *H*. *longicornis* at 6 dpi (Fig 1B), suggesting successful virus acquisition and replication through blood feeding.

We next evaluated the antiviral RNAi activity of these ticks by deep sequencing of small RNAs (sRNA-Seq). Consistent with the amplification of SFTSV, less vsiRNAs were detected in ticks at 2 dpi (Fig 1C, top panel). Of note, along with SFTSV replication, vsiRNAs with a clear peak length of 22 nt from both strands appeared in ticks at 6 dpi (Fig 1C, bottom panel, and Table 1). Additionally, compared to L and M segment of SFTSV, S segment produced more abundant vsiRNAs from both genome and antigenome (Fig 1C, bottom panel). Moreover, given that IFN-competent WT mice can also be used to establish a SFTSV infection model [64,65], we then performed a comparable experiment on WT mice and found that SFTSV also induced the production of canonical vsiRNAs in ticks feeding on BALB/c mice at 2 dpi (Fig 1D and 1E). These 21- to 23-nt vsiRNAs were predominantly clustered in the S segment from both polarities (Fig 1E), further indicating that the S segment is more accessible to tick RNAi machinery.

**Table 1:** Contents and properties of the small RNA libraries.

Library	Total reads (18-28nt)	miRNA^a^ (mature)	Virus reads (18-28nt)	Virus reads of 21- to 23-nt		
				Reads	% of total reads	% of all sizes
SFTSV: tick replete on A6 2dpi	5913507	693320	2	2	0.00%	100%
SFTSV: tick replete on A6 6dpi	9877943	1116656	740	661	0.01%	89.34%
SFTSV: tick replete on BALB/c 2dpi	12370294	807766	465	400	0.003%	86.02%
NoV: tick replete on C57BL/6 8dpi	32948742	3165401	161295	142326	0.43%	88.24%
SINV: tick replete on C57BL/6 6dpi	17731252	1570834	1185	1093	0.01%	92.24%
SINV: tick replete on C57BL/6 6dpi repeat	9480023	1609315	857	783	0.01%	91.37%
Tick mock	14617538	2741922	0	0	0.00%	/
NoV: tick microinjection 4dpi	9371844	1110467	3247	1982	0.02%	61.04%
NoVΔB2: tick microinjection 4dpi	6375814	861945	3952	2712	0.04%	68.62%
SINV: tick microinjection 5dpi	13313279	2424991	59912	56722	0.43%	94.68%
SINV: tick microinjection 5dpi repeat	12613473	3489225	62369	59357	0.47%	95.17%
SINV_NoV B2_:tick microinjection 14dpi	11794467	1698816	78284	56331	0.48%	71.96%
SINV_NoV B2_:tick microinjection 14dpi repeat	23686298	5006230	212570	146693	0.62%	69.01%
SINV_NoV mB2_:tick microinjection 14dpi	12057422	1559985	547327	507817	4.21%	92.78%
SINV_NoV mB2_:tick microinjection 14dpi repeat	20522622	4420167	1287076	1170252	5.70%	90.92%

^a^ Indicating the reads perfectly identical to *I*. *scapularis* mature microRNAs

NoV and SINV are frequently used model viruses to dissect antiviral RNAi [66]. We next expected to know the potential of those virus to dissect antiviral RNAi in ticks followed the same infection route (S1B and S1C Fig). After successful infection of *H*. *longicornis* ticks by NoV wild type (NoV WT) acquired from burdened mice (Fig 1F), vsiRNAs produced from ticks feeding on C57BL/6 mice were analyzed. These vsiRNAs are shown with a peak length of 22 nt and a slight hotspot in the sub-genomic RNA3 (Fig 1G). Although SINV exhibited weak replication in *H*. *longicornis* by blood feeding (Fig 1H), vsiRNAs from this library exhibited a length of 22 nt from both sense and antisense strands and 21- to 23-nt vsiRNAs mapped to the viral genome were approximately evenly distributed (Fig 1I and S2 Fig). Overall, these results demonstrate that SFTSV, NoV, and SINV are capable of inducing an antiviral RNAi response in *H*. *longicornis* under natural infection route.

### Whole-scale analysis of the antiviral immunity response against SFTSV infection in *H*. *longicornis*

**Fig 2:**
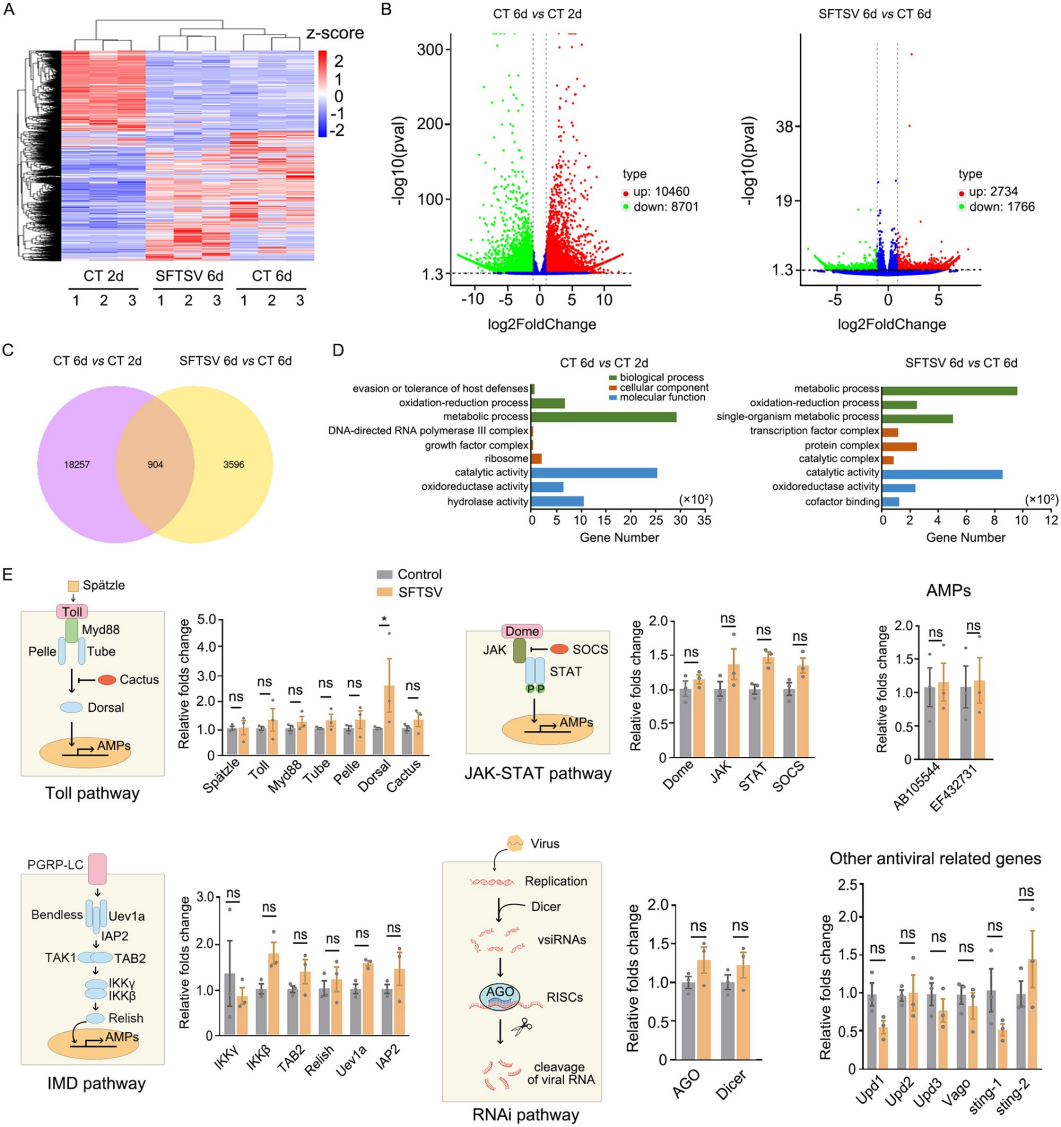
The status of immune response induced by SFTSV infection in *H*. *longicornis* ticks. (A) Hierarchical clustering of differentially expressed genes (DEGs) (log2|fold change|> = 1, p-value<0.05) from CT 2d, CT 6d and SFTSV 6d transcriptomes from *H*. *longicornis*. The scale represents z-score of gene FPKM (Fragments Per Kilobases Million). Nymphs had a blood meal for 2 days on mock-infected or SFTSV-infected suckling mice. For control (control treatment, CT) or infected ticks (SFTSV), the time was marked as 2d when ticks were removed from mice. If removed ticks were incubated for another 4 days, the time was marked as 6d. (B) Volcano plots show DEGs from CT 6d *vs* CT 2d and SFTSV 6d *vs* CT 6d. (C)Venn diagram of unique and common DEGs between CT 6d *vs* CT 2d and SFTSV 6d *vs* CT 6d. (D) Top Gene Ontology (GO) terms of up-regulated DEGs from CT 6d *vs* CT 2d and SFTSV 6d *vs* CT 6d. (E) The differential expression levels of genes from the primary innate immune pathways (Toll, IMD, JAK/STAT, RNAi, and other antiviral related genes) after control-treated and SFTSV infection at 6 days detected by RT-qPCR. The relative fold change of mRNA was calculated by ΔΔCt method and normalized using control-treated sample. Significance was determined by ANOVA with Dunn’s tests. ns: not significant. *P<0.05.

Besides the activation of antiviral RNAi, we would like to know the overall status of the immune response against SFTSV infection in *H*. *longicornis* through natural blood feeding. To this end, we performed high-throughput RNA sequencing analysis from the libraries of mock control and SFTSV-infected ticks at 2 or 6 dpi (designed as CT 2d, CT 6d, and SFTSV 6d). The *de novo* assembled transcriptomes were used as reference to perform a differential expression analysis between CT 2d, CT 6d, and SFTSV 6d. Hierarchical clustering of differentially expressed genes (DEGs) (log2|fold change|> = 1, p-value<0.05) show the similarity between these samples (Fig 2A).

The significant differentially expressed genes were determined from CT 6d *vs* CT 2d (Fig 2B, left, and S1 Table) and SFTSV 6d *vs* CT 6d (Fig 2B, right, and S1 Table). Overall, 10460 up-regulated and 8701 down-regulated DEGs were detected between CT 6d and CT 2d, and 2734 up-regulated and 1766 down-regulated DEGs were detected between SFTSV 6d and CT 6d. There are 904 common DEGs between CT 6d *vs* CT 2d and SFTSV 6d *vs* CT 6d, and 18257 and 3596 unique genes respectively (Fig 2C). The number of DEGs from CT 6d *vs* CT 2d was much more than that from SFTSV 6d *vs* CT 6d, which indicated that the blood feeding process had more prominent effect on tick gene transcription than the SFTSV infection. Based on sequence homology, up-regulated DEGs of CT 6d *vs* CT 2d and SFTSV 6d *vs* CT 2d were mainly classified into three Gene Ontology (GO) categories including biological processes, cellular components, and molecular function (Fig 2D). For CT 6d *vs* CT 2d, the term with most abundant genes was metabolic process (GO: 0008152), and the most significant enrichment term was evasion or tolerance of host defenses (GO: 0044415) (Fig 2D, left), which suggested that the metabolic response of ticks was heavily activated after the blood feeding, and meanwhile the immune response of ticks was simultaneously induced. For SFTSV 6d *vs* CT 6d, however, the most significant and gene-abundant term was metabolic process (GO: 0008152), and no GO term relative to immune response was enriched (Fig 2D, right).

Arthropod innate immunity is regulated by Toll, IMD, JAK/STAT, and antiviral RNAi pathways [27–33]. We then further focused on the differential expression level of genes from these four innate immune pathways. The results from quantitative PCR (qPCR) (Fig 2E) showed that the expression levels of some immune relative genes exhibited a slight up-regulation between mock and SFTSV infected ticks at 6 dpi, such as Dorsha in Toll pathway (Fig 2E).

Referring to recent tick genome resources [3], we selected the only Dicer gene in Fig 2E because one Dicer gene (HaeL12815) was annotated in the reference. There are at least 15 annotated Argonautes in *H*. *longicornis*. The selected AGO gene (termed Ago2-like gene later) in Fig 2E was determined from our assembled *H*. *longicornis* transcriptome by tblastn using *D*. *melanogaster* Ago2 gene sequence as query. The expression levels of these two selected RNAi relative genes showed no significant change upon virus infection (Fig 2E).

### Identification and characterization of Putative Dicer2-like protein in *H*. *longicornis*

Two Dicer enzymes, Dicer-1 and -2, are commonly present in fruit fly and mosquitoes. Dicer-1 is responsible for microRNA synthesis and Dicer-2 for siRNA pathways [67]. Jia. *et al* annotated one Dicer gene in *H*. *longicornis* depending on genome and transcriptome sequencing [3], which render us to further investigate whether a putative Dicer-1 and Dicer-2 like protein existent in *H*. *longicornis*.

To obtain the homologous gene coding sequences of *H*. *longicornis* Dicer-like proteins, we searched the assembled data of high throughput transcriptome sequencing from *H*. *longicornis* by BLAST using coding sequences of *D*. *melanogaster* Dicer-1 and Dicer-2 proteins as queries [67]. And we identified two corresponding homologous gene coding sequences in *H*. *longicornis*. We further cloned the complete open reading frames of these genes and validated by Sanger sequencing. To further determine the existence of these genes in *H*. *longicornis*, we mapped these two amplified coding genes back to two available *H*. *longicornis* genome databases [3,68], and found that 100% coverage and high identity were presented in *H*. *longicornis* genome (S2 Table).

**Fig 3:**
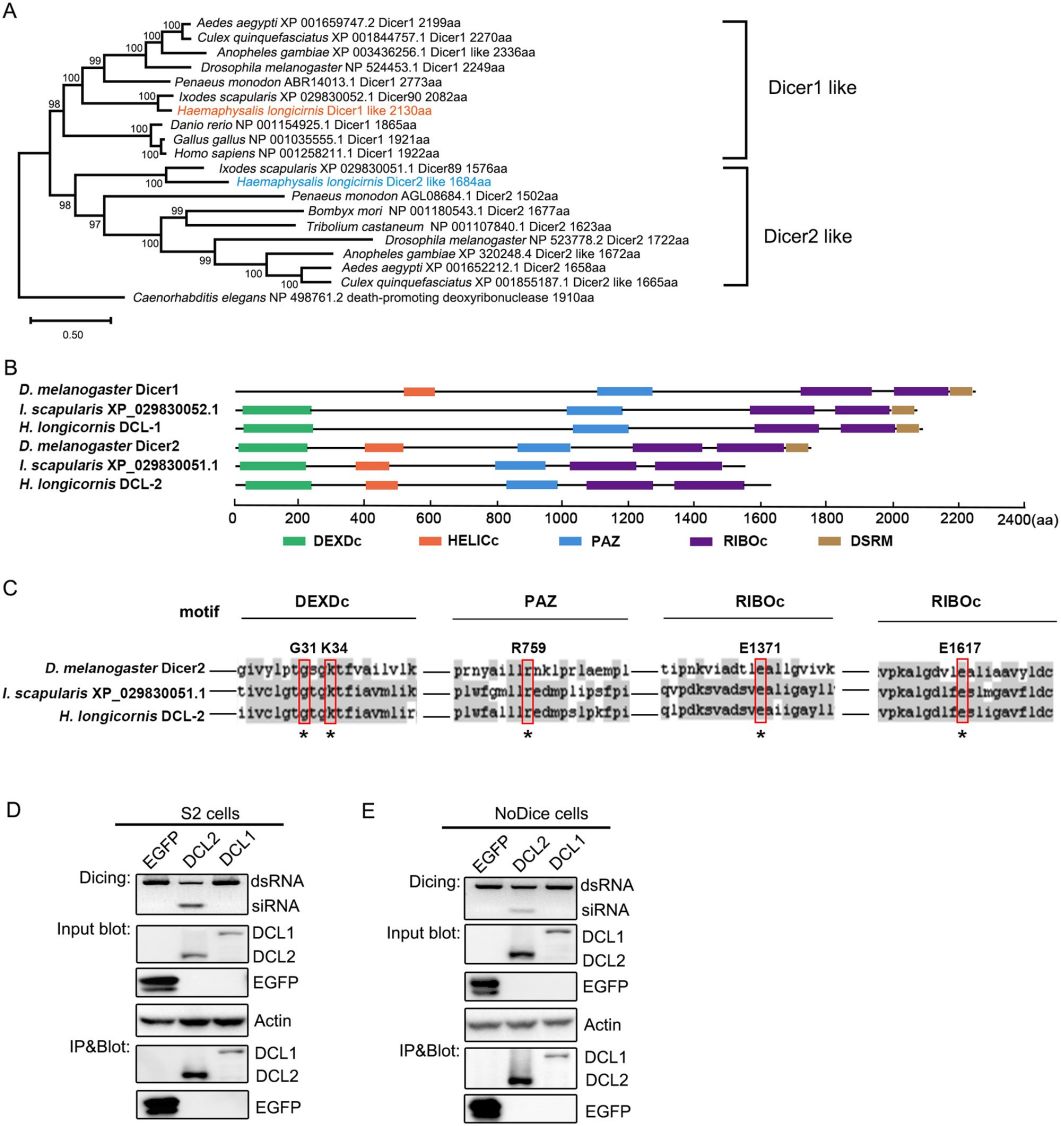
Identification and characterization of putative Dicer2-like protein in *H*. *longicornis* ticks. (A) Phylogenetic analysis of Dicer-like proteins. Maximum-likelihood (ML) trees were constructed using MEGA-X software with 1000 bootstrap based on the protein sequences of each Dicer-like member. *H*. *longicornis* Dicer-like proteins were highlighted. *C*. *elegans* NP_498761.2 was set as outgroup to root the tree. (B) Conserved domain structures of Dicer-like proteins from *D*. *melanogaster*, *I*. *scapularis* and *H*. *longicornis* predicted by SMART database. Domains are indicated by colored boxes. The scale bars at the bottom represent the length of proteins in amino acid (aa). (C) Comparison of conserved amino acids in Dicer2-like proteins. (D and E) *In vitro* dicing of a synthetic 200 bp dsRNA by ectopically expressing FLAG-tagged EGFP (as a control), DCL2, and DCL1 immune-precipitated from S2 cells (D) or NoDice 293T cells (E). Western blotting detection of input and immune-precipitated Flag-tagged EGFP, DCL2, and DCL1. The Dicer substrate and product small RNAs were detected by 3% agarose gel with GelRed staining. Endogenous β-actin as a loading control.

Phylogenetic analysis revealed that *H*. *longicornis* Dicer1-like protein (HlDCL-1) and *I*. *scapularis* Dicer XP_029830052.1 (Dicer90) grouped together in the cluster consisting of *Penaeus monodon*, mosquitos and *D*. *melanogaster* Dicer1 [52]. Meanwhile, *H*. *longicornis* Dicer2-like protein (HlDCL-2) and *I*. *scapularis* Dicer XP_029830051.1 (Dicer89) grouped together in the cluster consisting of *Penaeus monodon*, *Bombyx mori*, mosquitos and *D*. *melanogaster* Dicer2 (Fig 3A) [51]. HlDCL-1 is 2130 amino acids in length and share 73% identity with *I*. *scapularis* XP_029830052.1 (S3A Fig). The sequence contains DEXDc, PAZ, RIBOc and DSRM domains similar to *I*. *scapularis* XP_029830052.1, but lacks a HELICc domain compared to *D*. *melanogaster* Dicer1 (Fig 3B). Like other species with two Dicers, a second homologous Dicer2-like protein is shorter in length (1684 amino acids) than Dicer1-like protein and share 58% identity with *I*. *scapularis* XP_029830051.1 (S3B Fig). The sequence contains all the same domains as *I*. *scapularis* XP_029830051.1, but lacks a DSRM domain compared to *D*. *melanogaster* Dicer2 (Fig 3B). Notably, the essential amino acids for *D*. *melanogaster* Dicer2 activity, such as G31 and K34 in DEXDc domain, R759 in PAZ domain, E1471 and E1617 in RIBOc domain [67,69,70] were conserved in HlDCL-2 (Fig 3C).

To verify the function of HlDCL-2, we purified the ectopically expressing HlDCL-2 and HlDCL-1 enzymes from *Drosophila* S2 cells to process a synthetic 200bp dsRNA. Our results indicated that the HlDCL-2 but not the EGFP or HlDCL-1 could efficiently cleave the dsRNA into siRNA (Fig 3D). Our previous study demonstrates that the 22-nt vsiRNAs products can be detected in Dicer-deficient (NoDice) human 293T cells ectopically expressing fly Dicer-2 protein upon virus infection. So we purified the ectopically expressing HlDCL-2 from NoDice cells to process dsRNA. As expected, the purified HlDCL-2 from human cells was also a much better enzyme for processing dsRNA into siRNA (Fig 3E).

### Production of abundant vsiRNAs in *H*. *longicornis* ticks in response to virus replication initiated by intrathoracic injection

**Fig 4:**
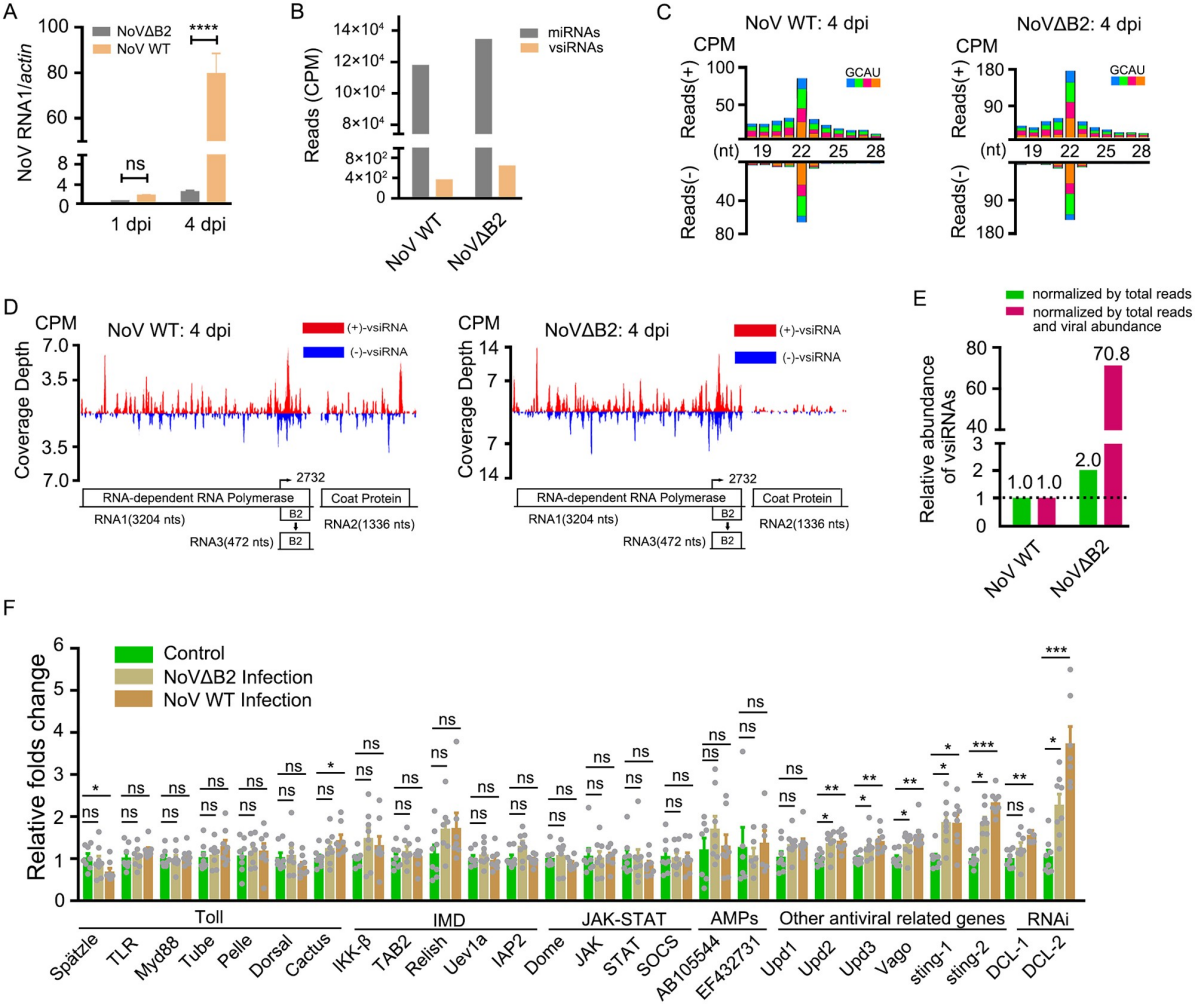
Production of abundant vsiRNAs in *H*. *longicornis* ticks infected by NoV through injection. (A) Viral RNA1 accumulation from ticks infected NoV WT or NoVΔB2 by microinjection at 1dpi and 4dpi was determined by RT-qPCR. (B) Reads of mature miRNAs and vsiRNAs from the small RNA-seq libraries of NoV WT or NoVΔB2-infected ticks at 4 dpi. (C) Size distribution of virus-derived small RNAs sequenced from ticks infected with NoV WT or NoVΔB2 at 4 dpi. (D) Genomic coverage depth of 21- to 23-nt vsiRNAs sequenced from NoV-infected or NoVΔB2-infected ticks. (E) Relative abundance comparison of 21- to 23-nt vsiRNAs sequenced from ticks infected with NoV WT or NoVΔB2 at 4 dpi. Read counts are normalized either by total 21- to 23-nt reads only (green bar) or by both total 21- to 23-nt reads and viral relative accumulation determined by RT-qPCR (red bar). (F) Relative folds change of immune related genes expression levels with NoV WT or NoVΔB2 infection at 4 dpi in *H*. *longicornis* ticks. For RT-qPCR analyses of viral accumulation, the viral RNA levels were calculated by ΔCt method and tick *β-actin* mRNA was used as the internal reference. For RT-qPCR analyses of innate immunity-related gene expression levels, ΔΔCt method was used. The data represent mean±SEM values of three biological replicates (10 nymphs per biological replicate) for viral accumulation detection and 8 biological replicates for gene expression level (3 nymphs per biological replicate). Significance was determined by ANOVA with Dunn’s tests. ns: not significant, *P<0.05, ** P<0.01, *** P<0.001. Read counts are shown as per million total 18- to 28-nt reads (CPM), and the 5’ terminal nucleotide of virus-derived small RNAs is indicated by different colors. Genomic coverage depth is indicated by the position of its 5’ terminal nucleotide. Sense strand-vsiRNAs are depicted in red, and antisense strand-vsiRNAs are depicted in blue. A schematic diagram representing the organization of NoV is presented.

Given the virus infection by natural blood feeding accompanies active nutritional metabolism of ticks, likely limiting the virus’s replication and accumulation. We chose the injection method for follow-up viral infection to further investigate the mechanism of antiviral RNAi in *H*. *longicornis*. The nymphal ticks were infected with NoV WT and NoVΔB2, in which the expression of B2 (VSR) is abolished with no effect on viral RdRp [60], through intrathoracic injection. We found that viral RNA accumulated progressively in both NoV WT and NoVΔB2-infected ticks over the time course, but NoV WT accumulated to a higher level than NoVΔB2 at 4 dpi (Fig 4A).

Given that NoV elicits robust replication in *H*. *longicornis*, we next examined whether vsiRNAs could be induced in ticks by sRNA-Seq. Indeed, NoV WT and NoVΔB2 both induced the production of vsiRNAs in ticks at 4 dpi (Fig 4B). The profiles of NoV-derived vsiRNAs exhibited characteristic features with a peak length of 22-nt and were divided approximately equally into positive and negative strands (Fig 4C). In order to dissect the genomic loci producing these vsiRNAs, vsiRNAs with 21- to 23-nt in length were mapped to the viral genome with perfect match setting. These vsiRNAs evenly distributed throughout the viral genome (Fig 4D). Notably, normalization of deep sequenced small RNAs identified more abundant vsiRNAs were induced by NoVΔB2 than those induced by NoV WT, particularly after normalization by viral abundance (Fig 4E), indicating that NoV B2 protein potentially counteract the production of vsiRNAs in *H*. *longicornis* as it performed in mammals [60–62]. These results suggest that the antiviral RNAi pathway is activated in *H*. *longicornis* upon NoV infection by injection and likely to be inhibited by the B2 protein.

We next investigated the expression patterns of innate immunity gene in response to NoV infection by injection. Our results demonstrated that most of the genes relative to Toll, IMD, and JAK/STAT pathway showed no significant change, and some genes such as sting-like genes (sting-1 and sting-2) significantly increased in ticks (Fig 4F). Notably, the expression level of identified HlDCL-2 also significantly increased along with the virus replication (Fig 4F).

### *In vivo* antiviral activity of the tick RNAi response

**Fig 5:**
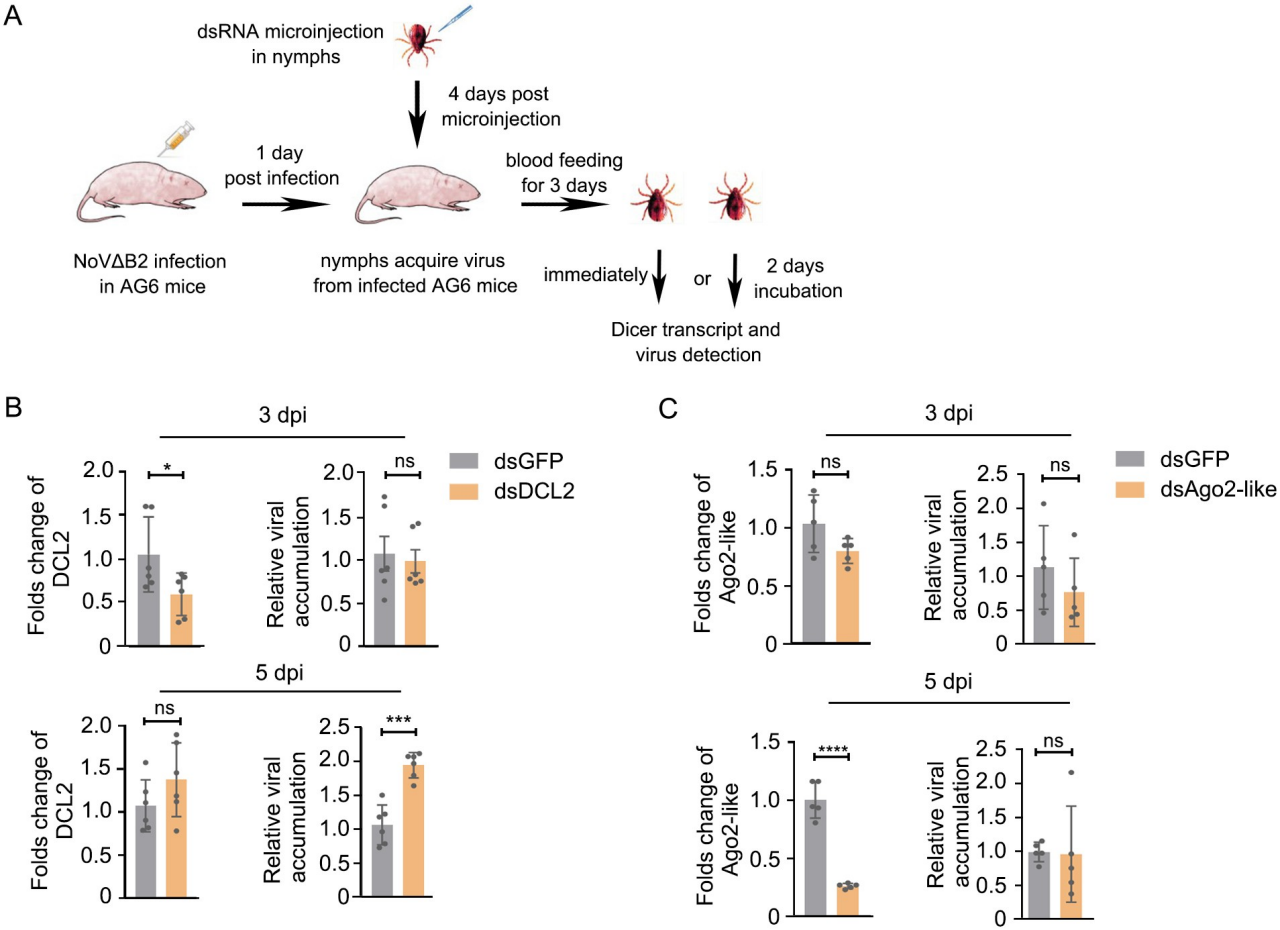
Putative Dicer2-like protein restricts viral infection in *H*. *longicornis*. (A) Schematic overview of dsRNA treatment in ticks. Briefly, AG6 mice were infected with NoVΔB2 by intraperitoneal injection. Nymphs injected with dsRNAs were allowed to parasitize AG6 mice for 3 days (designed as 3 dpi). After removing from the mice, half of nymphs was incubated for an additional 2 days (designed as 5 dpi). The HlDCL-2 and HLAgo2-like transcript levels and relative virus accumulations were determined by RT-qPCR. (B and C) Folds change of HlDCL-2(B) and HLAgo2-like(C) transcript level and viral accumulations in ticks with NoVΔB2 infection relative to dsGFP control. dsGFP: dsRNA targeting GFP, dsDCL2: dsRNA targeting HlDCL-2, dsAgo2-like: dsRNA targeting HlAgo2-like. For RT-qPCR analyses, ΔΔCt method was used, the data represents mean±SEM values of 5~6 biological replicates (3~5 nymphs per biological replicate). Significance was determined by the Mann-Whitney test. ns: not significant, *P<0.05, *** P<0.001, **** P<0.0001. Each experiment was repeated at least three times independently with one represented image shown.

Dicer-2 or Ago-2 loss-of-function mutation in insects usually results in enhanced accumulation of virus deficient in VSRs [35,36,66]. In this work, we identified a putative Dicer2-like protein in *H*. *longicornis*, which can efficiently process dsRNA into siRNA. To further dissect the role of Dicer2-like protein in antiviral RNAi, dsRNA-mediated knockdown of HlDCL-2 was conducted to assess its role on viral replication in ticks infected from a blood meal of NoVΔB2-infected AG6 mice (type I/II interferon receptor-deficient C57BL/6) (Fig 5A). Relative to dsRNA GFP control (dsGFP), dsRNA HlDCL-2 (dsDCL-2) treatment dramatically downregulated HlDCL-2 transcript levels at 3 dpi (Fig 5B). Although the viral accumulation was not different at 3 dpi, there was a significant increase after HlDCL-2 silencing compared with dsGFP control at 5 dpi (Fig 5B). The possible explanation is the HlDCL-2 protein level may be delayed compared to its mRNA level *in vivo*. These results suggest that the HlDCL-2 may be one of the core proteins of the antiviral RNAi pathway and is responsible for controlling viral infections.

To test knockdown of other components of the RNAi pathway that may lead to the same results, we selected one of the Argonaute proteins (Ago2-like protein) in *H*. *longicornis*. The dsRNA-mediated knockdown of Ago2-like was successful at 3 and 5dpi, but the viral accumulations were similar to the GFP controls (Fig 5C), suggesting this Ago2-like protein may not function in the antiviral RNAi pathway. Future studies are necessary to identify more functional components of the RNAi pathway.

### Expression of inversive correlation between vsiRNAs abundance and recombinant SINV load in *H*. *longicornis* ticks

A diverse range of viruses have evolved suppressors to counteract host antiviral RNAi defense mechanism [31,66]. For example, flock house virus (FHV) B2, NoV B2, and Influenza A virus (IAV) NS1 proteins are potent VSRs that inhibit the production of vsiRNAs by sequestering viral-derived dsRNA from host Dicer enzyme, which render the dysfunction of host antiviral RNAi [45,66]. Due to the absence of strong VSR activity in flies and mosquitos with SINV infections, recombinant SINV vector is commonly used to identify putative VSR or to characterize the function of a known VSR [41,57,71].

**Fig 6:**
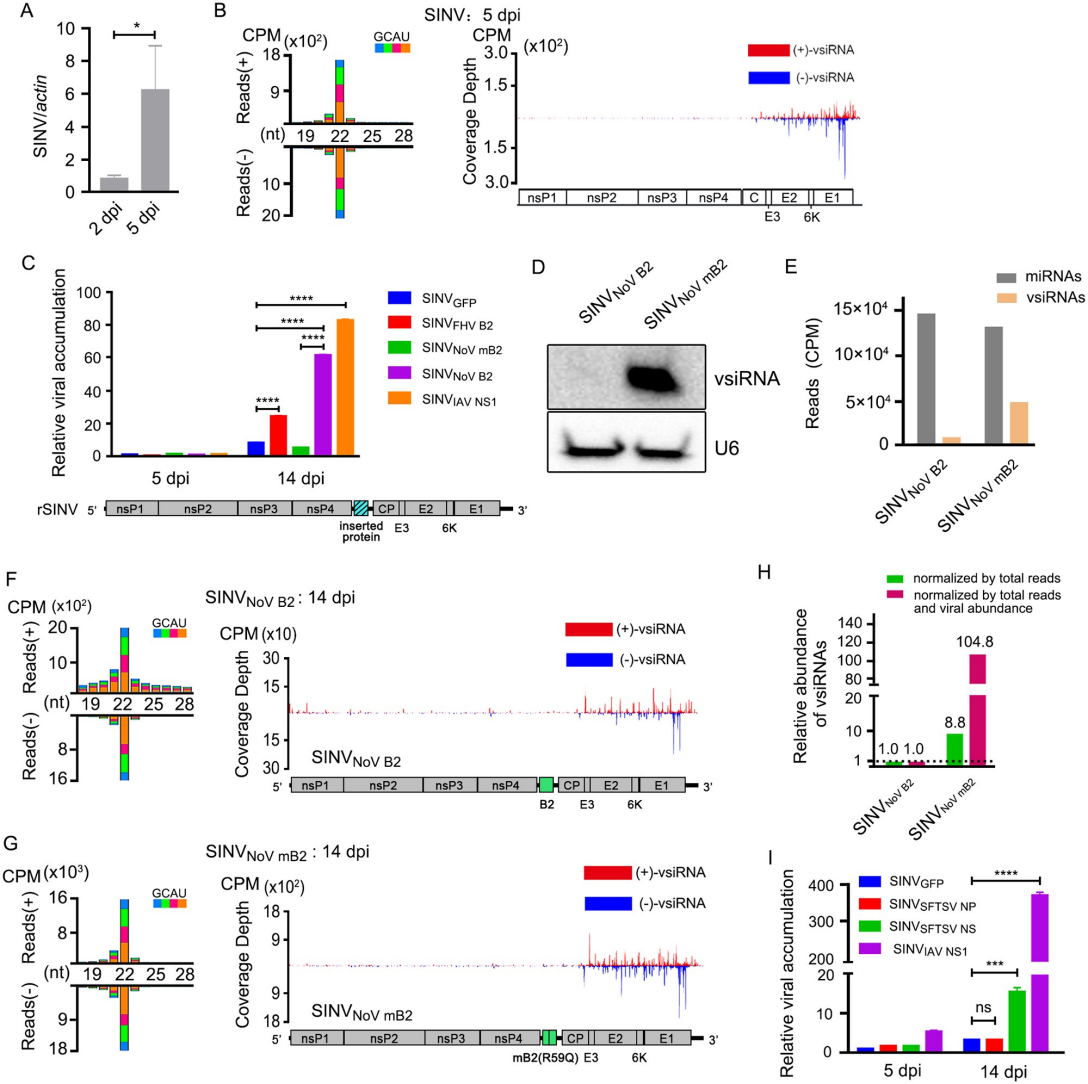
Infection of *H*. *longicornis* ticks with recombinant SINVs expressing heterologous VSR proteins. (A) SINV RNA levels in *H*. *longicornis* ticks infected with SINV by microinjection at 5dpi were determined by RT-qPCR. The viral RNA levels were calculated by ΔCt method and tick *β-actin* mRNA was used as the internal reference. (B) Size distribution of virus-derived small RNAs and genomic coverage depth of 21- to 23-nt vsiRNAs sequenced from ticks infected with SINV by microinjection at 5 dpi. (C) Relative viral accumulation determined by RT-qPCR in ticks after infection with SINV_GFP_, SINV_NoV B2_, SINV_NoV mB2_, SINV_FHV B2_, and SINV_IAV NS1_ by injection at 5dpi and 14 dpi. The viral RNA levels were calculated by ΔΔCt method. Tick *β-actin* mRNA as the internal reference. (D) Northern blotting detection of vsiRNAs in SINV_NoV B2_ and SINV_NoV mB2_ infected ticks by microinjection at 14 dpi. U6 as a loading control. (E) Reads of mature miRNAs and vsiRNAs in the library of SINV_NoV B2_ or SINV_NoV mB2_ infected ticks by microinjection at 14 dpi. (F and G) Size distribution of virus-derived small RNAs and genomic coverage depth of 21–23 nt vsiRNAs sequenced from ticks after infection with SINV_NoV B2_ (F) and SINV_NoV mB2_ (G) by microinjection at 14 dpi. (H) Relative abundance comparison of 21- to 23-nt vsiRNAs sequenced from ticks infected with SINV_NoV B2_ and SINV_NoV mB2_ at 14 dpi. Read counts were normalized either by total 21- to 23-nt reads only (green bar) or by both total 21- to 23-nt reads and viral relative accumulation determined by RT-qPCR (red bar). (I) Relative viral accumulations determined by RT-qPCR in ticks after infection with SINV_GFP_, SINV_SFTSV NP_, SINV_SFTSV NS_, and SINV_IAV NS1_ by microinjection at 14dpi. The viral RNA levels were calculated by ΔΔCt method. Tick *β-actin* mRNA as the internal reference. Error bars indicate the SEM of three biological replicates (5 nymphs per biological replicate). dpi: days post injection. Significance was determined by the Mann-Whitney test in (A) and ANOVA with Dunn’s tests in (C) and (I). ns: not significant, *P<0.05, *** P<0.001, ****P<0.0001. Read counts are shown as per million total 18- to 28-nt reads (CPM) and the 5’ terminal nucleotide of virus-derived small RNAs is indicated by different colors. Genomic coverage depth of 21-to 23-nt vsiRNAs is indicated by the position of its 5’ terminal nucleotide. Sense strand-vsiRNAs are depicted in red, and antisense strand-vsiRNAs are presented in blue. A schematic diagram representing the genomic organization of rSINV is shown.

We first investigate whether SINV has the competency to replicate and induce antiviral RNAi in *H*. *longicornis* by injection. The abundance of SINV increased significantly at 5 dpi compared to that at 2 dpi after injection infection (Fig 6A). The replication of SINV in ticks by injection was extremely higher than the virus by natural blood feeding (Figs 1H and 6A). Consistent with viral replication, abundant accumulation of vsiRNAs with a peak length of 22 nt from both strands were induced at 5 dpi (Fig 6B and S4 Fig). To visualize the distribution of vsiRNAs in the viral genome, 21- to 23-nt vsiRNAs were mapped to the viral genome, and they distributed throughout the genome and antigenome in a cold and hot spot pattern (Fig 6B and S4 Fig). Interestingly, the genomic loci were significantly clustered towards to the 3’ end of the SINV genome, corresponding to the sub-genomic RNA encoding the structural proteins, which was significantly different from the pattern obtained by blood feeding. Overall, these data suggested that consistent with its performance in *Drosophila* [35,55,56] and mosquitos [40–42], SINV could be utilized to decipher the mechanism of antiviral RNAi in ticks.

Based on the performance of SINV in ticks, we next want to further dissect antiviral RNAi of tick in the view of VSRs. *H*. *longicornis* ticks were infected with equivalent titers of SINV recombinant with NoV B2, NoV mutant B2 (mB2, VSR-dysfunctional B2 with a single Arg to Gln mutation at 59 of B2, which abolishes VSR activity), FHV B2, IAV NS1, and GFP (Fig 6C, bottom of the panel). Compared with *H*. *longicornis* infected with SINV_GFP_, ticks infected with the recombinant SINV (rSINV) expressing the heterologous VSR proteins, B2 and NS1, significantly enhanced the virus accumulation at 14 dpi (Fig 6C). Notably, the replication level of rSINV expressing the NoV mB2 protein was dramatically reduced in *H*. *longicornis* ticks (Fig 6C).

To further elucidate whether vsiRNAs modulated rSINV replication, we performed Northern blotting for comparing the abundance of vsiRNAs from SINV_NoV B2_ and SINV_NoV mB2_ samples. We identified more abundant vsiRNAs were induced by SINV_NoV mB2_ than those induced by SINV_NoV B2_ (Fig 6D). We next analyzed sRNAs-Seq of relevant infected ticks (Fig 6E). The results revealed that although the accumulation of a dominant peak of 22-nt vsiRNAs from both polarities produced in ticks infected with both SINV_NoV B2_ (Fig 6F and S5A Fig) and SINV_NoV mB2_ (Fig 6G and S5B Fig), vsiRNAs from SINV_NoV mB2_ were more abundant than that from SINV_NoV B2_ (Fig 6H and S5C Fig). This trait was even pronounced when the abundance of vsiRNAs was normalized to viral replication (Fig 6H and S5D Fig). Based on these results, we conclude that NoV B2 protein suppresses antiviral RNAi targeting SINV in *H*. *longicornis* ticks.

### NS protein of SFTSV may function as a potential VSR

Viral protein often acts as a multi-functional effector to antagonize host immune response and many IFN-antagonistic viral proteins are known to act as VSR as well [66]. Previous studies suggested that NS protein of SFTSV acted as a critical virulence factor to block the interferon response in mammals and that NP protein of SFTSV was essential for viral RNA encapsidation through RNA binding [26,72,73]. To test potential VSR activity of NS and NP proteins of SFTSV, recombinant SINV strains containing these two proteins were constructed and rescued (S6 Fig), respectively. Taking advantage of the rSINV strategy, we found that NS not NP could facilitate the replication of rSINV in *H*. *longicornis* ticks in a similar way as the IAV NS1, although exhibited a more modest activity (Fig 6I). This result suggests that the NS protein may function as a potential VSR among SFTSV infection and shed light on the identification of VSR in vertebrate-infecting bunyaviruses.

## Discussion

Short of an effective virus research system greatly hindered the study on antiviral immunity mechanisms in *H*. *longicornis* [3,4,32]. Here, we establish a novel model for virus-vector-mouse experiments with longhorned ticks. We tested the replication of model arthropod-borne RNA viruses in *H*. *longicornis* after infection by either natural blood feeding or/and injection. Consistent with the performance of NoV and SINV as models to elucidate antiviral RNAi in arthropods and mammals, both viruses induce abundant levels of vsiRNAs in *H*. *longicornis* after infections, suggesting NoV and SINV are valuable models to explore the mechanism of antiviral RNAi in ticks. Meanwhile, to gain insight into the response of tick to virus infection, the transcriptomes of longhorned ticks infected with the SFTSV were analyzed in this study, providing the first differential expression analysis of longhorned tick’s antiviral responses.

In flies and mosquitos, viral derived dsRNA is recognized and processed into a pool of siRNA duplexes predominantly 21-nt in length by Dicer-2 [45,66]. Here, our study shows that vsiRNAs induced by different viral infection in *H*. *longicornis* share a common feature of a peak length of 22-nt, which is consistent with the feature noted in *Ixodes scapularis* ticks upon flavivirus infections [52,53]. For NoV infection in ticks, the genomic distribution of NoV-derived vsiRNAs by the injection is similar to that produced from the natural blood feeding, although more abundant vsiRNAs are produced from the latter. Previous studies show that vsiRNAs produced from mosquitos and flies after infection with SINV are approximately evenly distributed throughout the viral genome [41,74]. Intriguingly, our results show that the vsiRNAs produced in SINV infected ticks by blood feeding exhibit a similar pattern as in mosquitos and flies. In contrast, those produced by injection are primarily bias towards to the SINV subgenomic region, which is responsible for the production of structural proteins. This is the first time that the same virus has been found to produce distinguished patterns of vsiRNAs in ticks through two different routes of infection.

Blood feeding is the natural route of arbovirus entry, and arthropod midgut is the first encounter to invading viruses. In contrast, injection is an artificial method by directly injecting arboviruses into the thoracic cavity, which bypass the midgut barrier [75,76]. Comparing the two infection routes, we detected more robust replication of SINV by injection than blood feeding (Figs 1H and 6A). Moreover, ticks are obligate hematophagous arthropods which need blood feeding and digestion to provide nutrition and energy for their metamorphosis [1]. So, virus acquisition from blood meal may accompanies with activated nutritional metabolism and tick development. Of note, some host blood constituents or metabolites from blood digestion, such as cytokines [77], growth factors [78,79], serum iron [80], immunoglobulins against viral protein [81] and low-density lipoprotein [82], remain physiological and immunological activity, which may modulate the immune response of the vectors, and then influence the virus infections.

In this work, we provide unique insight into the interaction between SFTSV and its natural vector giving that antiviral RNAi is induced by viral infection. Our results suggest that antiviral RNAi functions as an evolutionarily conserved immune defense mechanism in *H*. *longicornis* ticks *in vivo*. With the completion of high-quality genomic resources of six ixodid ticks [3], we may discover more immune-related genes of tick. Interestingly, according to these genome resources, only one Dicer-like protein is annotated in *H*. *longicornis* (HaeL12815, close to HlDCL-1) and *Rhipicephalus sanguineus* (Rsan23305), respectively [3]. In contrast, four Dicer-like proteins are annotated in *Dermacentor silvarum* [3]. Moreover, the diversity of Argonaute proteins are more complicated than Dicer-like proteins in ticks (15 annotated Argonautes in *H*. *longicornis*) [3]. In the present research, we identified two putative tick Dicer-like proteins, HlDCL-1 and HlDCL-2, in the *H*. *longicornis* ticks. The HlDCL2 but not the HlDCL1 can efficiently process dsRNA into siRNA. For tick Argonaute proteins, the Ago2-like gene selected in this study may not function in the antiviral RNAi pathway. Therefore, it is critical to combine multiple identification methods to determine more RNAi-relative genes of ticks.

To counteract the potent antiviral RNAi machinery in arthropods, viruses have evolved suppressors to antagonize this pathway [38,39,66,83]. The outcome of this interaction generally renders persistent infection in arthropods, thus allowing them to serve as an effective vector for viral transmission [84]. B2 proteins of NoV and FHV are well-documented suppressors that sequester dsRNA from processing by Dicer and siRNAs from incorporation into RISC [46,85]. Studies in flies, mosquitos, and mammals have demonstrated that NoV or FHV B2 proteins can significantly combat the production of vsiRNAs, which are inversely correlated with elevated viral burden and increased mortality [36,41,60]. In this work, we demonstrate that functional intact B2 but not mutant B2 acts as a suppressor of RNAi to antagonize the production of vsiRNAs in ticks. This notion is confirmed by the relative abundance of vsiRNAs in ticks infected with NoV WT and NoVΔB2 through injection. Utilizing recombinant SINV system, we provide evidence that SFTSV NS protein may function as a putative VSR in tick vector. Notably, NS protein encoded by a diverse range of viruses in the order *Bunyavirales* have been identified as a suppressor of RNAi. For example, NSs protein of *T*omato spotted wilt virus (TSWV) has been identified as a suppressor in plants [86]. NSs protein of La Crosse virus (LACV) has been described to counteract the effects of short interfering RNA [87]. However, one report indicated that in mosquito cells overexpressed LACV NSs was unable to inhibit RNAi against Semliki Forest virus [88]. NS3 protein of Rice stripe virus (RSV) and rice hoja blanca virus (RHBV) also exhibited suppressor activity of RNAi [89]. The performance of NS protein of SFTSV in ticks suggested that the RNAi suppressor activity of non-structural protein may be a general feature within the order *Bunyavirales*. In addition, existed evidence has indicated TSWV NS protein is capable of inhibiting antiviral RNAi both in plant and arthropod host. Notably, SFTSV can be circulating in nature by transmission of infectious virions between ticks and mammals. Therefore, whether SFTSV NS protein elicits VSR activity in mammals needs further investigation.

## Materials and methods

### Ethics statement

All the animal experimental protocols used in this study were approved by the IACUC (Institutional Animal Care and Use Committee) of Fudan University and performed in strict accordance with IACUC guidelines.

### Cell culture

Baby hamster kidney cells (BHK) were purchased from the American Type Culture Collection (ATCC). The NoDice Human embryonic kidney (293T) cell line was gift from Dr. B. Cullen. The *Drosophila* Schneider 2 (S2) cell line was gift from Dr. Y. Qi. BHK and No Dice 293T cells were cultured in Dulbecco’s modified Eagle’s medium (DMEM) supplemented with 10% heat-inactivated fetal bovine serum and 1% antibiotic/antimycotic at 37°C with 5% CO_2_, and S2 cells were cultured in Schneider’s Drosophila Medium (Gibco) with 10% heat-inactivated fetal bovine serum and 1% antibiotic/antimycotic at 28°C.

### Mice and ticks

BALB/c and C57BL/6 wild-type mice were purchased from Shanghai SLAC Laboratory Animal Co., Ltd. C57BL/6 mice deficient in type I interferon receptor (A6) were purchased from Cyagen Biosciences (Suzhou, China). C57BL/6 mice deficient in type I/II interferon receptor (AG6) were kindly provided by Dr. Q. Len. All mice were bred in a pathogen-free barrier at Fudan University, Shanghai. *H*. *longicornis* ticks originally collected from Yunnan Province of China were maintained at the insectary of Fudan University. Ticks were maintained at 26 °C with 85% humidity under a 12/12-h light/dark photoperiod.

### Viruses

NoV WT and mutant NoVΔB2 strains used in this study were previously described [60]. SFTSV was kindly provided by Dr. W. Shi. SINV were rescued from the plasmid of pSVN1 gifted from Dr. C.M. Rice. Briefly, the plasmid was first linearized with *Xhol* I (New England BioLabs) and then *in vitro* transcribed using a SP6 mMESSAGE mMACHINE kit (Ambion) to produce naked SINV genomic RNAs. Those RNAs were subsequently purified by TRIzol reagent (Invitrogen) and transfected into BHK cells using a TransIT-mRNA Transfection Kit (Mirus Bio, WI). Viruses were harvested and titrated by plaque assay as previously described [90].

### Construction and rescue of recombinant SINV

The plasmid named pTE/5’2J/GFP (SINV expressing EGFP) and pTE/5’2J were gifts from C.M. Rice. In pTE/5’2J, the open reading frame (ORF) of proteins of interest were flanked by *Apa I* and *Xba I* sites (New England BioLabs) and then inserted into the multiple cloning site (MCS) downstream of *nsp4* gene. Transcription of the inserted ORF was controlled by the duplicated subgenomic promoter sequence ahead of MCS. Recombinant SINV expressing NoV B2, NoV mB2 (single G to A substitution at nucleotide 2919 of RNA1 that abolishes the dsRNA binding property of B2 but without affecting viral RdRp), FHV B2, IAV NS1, SFTSV NS, and SFTSV NP were constructed, rescued and titrated as mentioned [91].

### Infection of mice and ticks

1. Ticks infected with SFTSV by blood feeding: 6- to 8-day-old A6 or BALB/c suckling mice were intraperitoneally injected with SFTSV of 10^4^ plaque-forming units (PFU) in 50-μL total volume of inoculum. 24 hours post SFTSV infection in mice, nymphs were allowed to feed on SFTSV-infected A6 or BALB/c for 2 days (marked as 2dpi for ticks), then collected and incubated for an additional 4 days (Infected ticks from A6, marked as 6dpi) or 8days (Infected ticks from BALB/c, marked as 10 dpi).
2. Ticks infected with NoV WT by blood feeding: 6- to 8-day-old C57BL/6 suckling mice were intraperitoneally injected with NoV WT preparation containing 7x10^6^ copies of genomic RNA1 from the titrated set of stocks. 24 hours post NoV WT infection in mice, nymphs were allowed to feed on NoV WT-infected C57BL/6 for 2 days, then collected and incubated for an additional 6 days (marked as 8 dpi).
3. Ticks infected with SINV by blood feeding: 6- to 8-day-old C57BL/6 suckling mice were intraperitoneally injected with SINV of 50 PFU in 50-μL total volume of inoculum. 24 hours post SINV infection in mice, nymphs were allowed to feed on SINV-infected C57BL/6 for 2 days, then collected and incubated for an additional 4 days (marked as 6 dpi).
4. Ticks infected with NoV WT or NoVΔB2 by microinjection: nymphs were injected intrathoracically with 10 nl virus solution [NoV WT (3.6x10^3^ genome RNA copies), NoVΔB2 (3.6x10^3^ genome RNA copies)]and collected at 1dpi and 4dpi. Mock control was injected with DMEM solution.
5. Ticks infected with SINV by microinjection: nymphs were injected intrathoracically with 10 nl SINV virus solution (5 PFU) and collected at 2 dpi and 5 dpi.
6. Ticks infected with rSINV by microinjection: nymphs were injected intrathoracically with 10 nl rSINV virus solution (5 PFU) and collected at 5 dpi and 14 dpi.

The microinjection was performed by Nanoinject III (Drummond Scientific Company, Broomall, PA).

### Reverse transcription and real-time qPCR

Total RNA was isolated from *H*. *longicornis* ticks tissues using TRIzol reagent (Invitrogen) following the manufacturer’s protocol. Complementary DNA (cDNA) was generated from 1 μg total RNA using HiScript III 1st Strand cDNA Synthesis Kit (+gDNA wiper) (Vazyme). Real-time qPCR was performed with the ChamQ Universal SYBR qPCR Master Mix (Vazyme) using diluted cDNA (1:10). All experimental operations were according to the manufacturer’s protocol. The primers used for this analysis were listed in S3 Table.

### Analysis of the primary innate immune pathways in *H*. *longicornis*

Since the annotation information of genes from the primary innate immune pathways in *H*. *longicornis* is limited, the genes we firstly selected based on the annotated genes released in S3 Table of the reference [3] with conserved domains determined by blast using *D*. *melanogaster* homologous genes as queries, including Spätzle:HaeL01656, Toll:HaeL11092, MyD88:HaeL14441, Tube:HaeL19125, Pelle:HaeL29011, Dorsal:HaeL18051, Cactus:HaeL17816, IKKγ:HaeL01701, IKKβ:HaeL06586, TAB2:HaeL28415, Relish:HaeL00576, Uev1a:HaeL02814, IAP2:HaeL20203, Dome:HaeL04169, JAK:HaeL07502, STAT:HaeL06063, SOCS:HaeL28514, and Dicer:Hael12815. The selected AGO gene was determined from our assembled *H*. *longicornis* transcriptome by tblastn using *D*. *melanogaster* Ago2 gene sequence as query, which was homologous with Hael22487. We designed primers based on these sequences and detected these genes’ expression levels by RT-qPCR. For other relative antiviral genes, including unpaired genes, vago, and sting, which were not annotated in S3 Table of the reference [3], we obtained *H*. *longicornis* homologous sequences from our assembled *H*. *longicornis* transcriptome by tblastn using these genes’ coding sequences of *D*. *melanogaster* as queries and selected one with top read counts of candidates as the detection object. For AMPs genes, there are three reported *H*. *longicornis* AMPs in GenBank database (AB105544, EF432731 and EF432732). Two (AB105544 and EF432731) with most mapped reads counts in our assembled transcriptome were selected for analysis.

### Cloning of full-length Dicer and phylogenetic analysis

To obtain the full-length sequence of *H*. *longicornis* Dicer, we designed the forward and reverse primer locating in the 5’-UTR and 3’-UTR of the gene respectively according to the sequence information obtained from resembled transcriptome reads. 4 μg total RNA was applied to synthesis the first-strand cDNA using oligo(dT)_20_ with SuperScript III reverse transcriptase (Invitrogen) following the manufacturer’s protocol. Full-length *H*. *longicornis* Dicer was PCR amplified from a 4 μL volume of cDNA templet using LA Taq DNA polymerase (TAKARA) according to the manufacturer’s protocol. The specific amplicon of ~6400 base pairs for HlDCL-1 and ~5000 base pairs for HlDCL-2 were sequenced using Sanger sequencing method. The obtained DNA sequence was translated into protein sequence using SnapGene software and then align with Dicer protein sequences of other species. These protein sequences were retrieved from NCBI database: *D*. *melanogaster* Dicer1 (NP_524453.1), *D*. *melanogaster* Dicer2 (NP_523778.2), *H*. *sapiens* Dicer (NP_803187.1), *C*. *elegans* Death-promoting deoxyribonuclease (NP_498761.2), *I*. *scapularis* Dicer89 (XP_029830051.1), *I*. *scapularis* Dicer90 (XP_029830052.1), *Aedes aegypti* Dicer2 (XP_001652212.1), *Aedes aegypti* Dicer1 (XP_001659747.2), *Anopheles gambiae* Dicer1 (XP_003436256.1), *Anopheles gambiae* Dicer2 (XP_320248.4), *Culex quinquefasciatus* Dicer1 (XP_001844757.1), *Culex quinquefasciatus* Dicer2 (XP_001855187.1), *Danio rerio* Dicer1 (NP_001154925.1), *Gallus gallus* Dicer1 (NP_001035555.1) *Penaeus monodon* Dicer2 (AGL08684.1), *Penaeus monodon* Dicer1 (ABR14013.1), *Bombyx mori* Dicer2 (NP_001180543.1), *Tribolium castaneum* Dicer2 (NP_001107840.1). Conserved domain was searched using the online servers of SMART (http://smart.embl-heidelberg.de/) and manually checked. Phylogenetic analysis of full-length Dicer protein from these organisms was performed using MEGA-X software with maximum-likelihood method.

### Western and Northern blotting analyses

Western and Northern blotting analysis were performed as described previously [92]. Anti-Flag mAb was purchased from GenScript (A01428-100). The original blots were showed in S7 Fig. Sequences of probes used for Northern blotting to detect vsiRNA: CACACAGCGATACGATAAAGTT; ATCAGTTCCTCGGAAGTACATC; GGCTGTTCTCACTGTCGCAAAA.

### *In vitro* assay of Dicer-like proteins activity

pAc5.1-N-3xflag-EGFP, pAc5.1-N-3xflag-DCL2, and pAc5.1-N-3xflag-DCL1 were transfected into S2 cells using Insect Transfection Reagent (Mirus, MIR6100). pCMV-N-3xflag-EGFP, pCMV-N-3xflag-DCL2, and pCMV-N-3xflag-DCL1 were transfected into NoDice 293T cells using Lipofectamine 2000. Briefly, cells were seeded in a 6-well plate one day before transfection. 48 hours after transfection, the cells were harvested and lysed in cell lysis buffer (CST). The FLAG-tagged proteins were retrieved using FLAG beads (GenScript) according to the manufacturer’s instructions. For dicing assays, FLAG-tagged proteins with beads were incubated with 30ug 200bp dsRNA in 200ul dicing buffer [250mM NaCl, 30mM HEPES, 0.05mM EDTA, 2.5mM MgCL2, 1mM DTT, 5% glycerol, 1.5mM ATP] for overnight at 30°C (from S2 cells) or 37°C (from NoDice 293T cells). 10ul dicing buffer of each sample was loaded onto 3% agarose gels and Visualize RNA by staining with GelRed.

### RNA interference

Silencing of *H*. *longicornis* DCL2 and Ago2-like transcript was achieved by dsRNA-mediated RNA interference. Briefly, target region for DCL2 and Ago2-like knock-down was PCR amplified from obtained HlDCL-2 full-length amplicon or cDNA of ticks using specific primers containing T7 promoter sequence as shown in S3 Table. The sequence of Ago2-like with completed conserved domains was one of the candidates determined from our assembled *H*. *longicornis* trancriptome by tblastn using *D*.*melanogaster* Ago2 protein sequence as query. The amplified PCR fragment was then purified and used to synthesize dsRNA using the MEGAscript T7 kit (Invitrogen). DsRNA targeting GFP was synthesized following the same procedure and used as the control. For injection of dsRNA, 10 nl dsRNA solution (1500ng/μl) was microinjected into the thorax of nymphs. The injected nymphs recovered for 4 days and then acquired virus by feeding on one day post NoVΔB2-infected suckling AG6 mice for 3 days (marked as 3dpi). Part of collected nymphs were incubated for an additional 2 days (marked as 5dpi). 3dpi and 5dpi ticks were processed for assessment of knockdown efficiency and viral accumulation by RT-qPCR.

### RNA-seq and data analysis

Total RNA was extracted from infected or control ticks using TRIzol reagent. The integrity of the purified RNA was analyzed by the Agilent 2200 Electrophoresis Bioanalyzer System (Agilent Technologies). Enrichment of poly (A)-RNA preparation for RNA Sequencing was performed using NEBNext Poly (A) mRNA Magnetic Isolation Module (NEB) kit. The cDNA libraries were constructed for each pooled RNA sample using the NEBNext Ultra Directional RNA Library Prep Kit for Illumina according to the manufacturer’s instructions. The products were purified and enriched by PCR to create the final cDNA libraries and quantified by Agilent2200. The tagged cDNA libraries were pooled in equal ratio and used for 150 bp paired-end sequencing in a single lane of the Illumina HiSeqXTen. Clean reads were obtained from the raw reads by removing the adaptor sequences, reads with > 5% ambiguous bases (noted as N) and low-quality reads containing more than 20 percent of bases with qualities of < 20. *De novo* assembly of transcriptome was accomplished based on clean reads from all libraries using Trinity with default parameters [93]. Gene function was annotated based on the following databases: nr (NCBI non-redundant protein sequences), nt (NCBI non-redundant nucleotide sequences), pfam (Protein family), KOG/COG (Clusters of Orthologous Groups of proteins), Swiss-Prot (A manually annotated and reviewed protein sequence database), KO (KEGG Ortholog database), GO (Gene Ontology). Differential expression analysis of any two groups was performed using the DESeq R package [94] and the differentially expressed genes between samples were identified by |log2fold change|> 1 and p-value< 0.05. Hierarchical clustering of differentially expressed genes was performed by pheatmap R package and Volcano plots graph was performed by ggplot2 R package. Gene Ontology (GO) analysis was applied to analyze the primary functions of the differentially expressed genes [95]. Fisher’s exact test was applied to identify the significant GO categories.

### Statistical analysis

Differences in this study were statistically analyzed using GraphPad Prism (version 8, La Jolla, CA). Two-tailed non-parametric t-test was performed to compare statistical significance. The viral RNA levels were calculated by ΔCt method and tick *β-actin* mRNA was used as the internal reference. For RT-qPCR analyses of innate immunity-related gene expression levels, ΔΔCt method was used. P value<0.05 was considered significant. All experiments were biologically repeated two or three times.

### Construction of small RNA libraries

Total RNA extracted with TRIzol regent in this study was used for the construction of small RNA libraries via the method that employs the 5’ monophosphate of small RNAs as described previously using the TruSeq Small RNA Sample Preparation Kit of Illumina (San Diego, CA) [92].

### Deep sequencing and bioinformatic analysis of small RNAs

Libraries of small RNAs were cloned from the RNA samples and sequenced by Illumina HiSeq 2000/2500. Adapter sequences were removed from small RNA reads, and reads were mapped to the reference virus genome using Bowtie 1.1.2 software with perfect match settings. All of the references used were downloaded from web sources. Subsequent bioinformatics analysis of virus-derived small RNAs was performed out using in-house Perl scripts as described previously [92]. Read counts are shown as per million total 18- to 28-nt reads (CPM) in the figures, and the 5’ terminal nucleotide of virus-derived small RNAs is indicated by different colors. Genomic coverage depth is indicated by the position of its 5’ terminal nucleotide. Sense strand-vsiRNAs are depicted in red, and antisense strand-vsiRNAs are depicted in blue. The reference sequences used in this study are either identical with those described previously or listed below:

1. NoV RNAs 1 and 2: AF174533.1 and AF174534.1
2. NoVΔB2 RNAs 1 and 2: the same as NoV except for 3 substitutions in RNA1: U2745C, U2754C and C2757G.
3. SINV: J02363.1
4. SFTSV: KX641917.1 for segment S, KX641913.1 for segment M and KX641909.1 for segment L.
5. Mature miRNAs: miRbase 21 (http://www.mirbase.org/).

## Supporting information

S1 Fig Virus accumulation in mice. (A) The replication levels of SFTSV in the hind limb of A6 and BALB/c suckling mice at 3 dpi were determined by RT-qPCR. (B) The replication levels of NoV in the hind limb of BALB/c and C57BL/6 suckling mice at 3 dpi were determined by RT-qPCR. (C) The replication levels of SINV in the hind limb of BALB/c and C57BL/6 suckling mice at 3 dpi were determined by RT-qPCR. Total RNA was extracted from the hind limb muscle tissue of mice using TRIzol reagent. The viral replication level was calculated by ΔCt method. *β-actin* mRNA as the internal reference.
(TIF)

S2 Fig Small RNA sequencing of ticks infected with SINV by feeding on mice, a repeat of Fig 1I. Size distribution of total reads (left), virus-derived small RNAs (middle) and genomic coverage depth of 21- to 23-nt vsiRNAs (right) sequenced from ticks infected with SINV by feeding on C57BL/6 mice at 6dpi. Read counts are shown as per million total 18- to 28-nt reads (CPM) and the 5’ terminal nucleotide of virus-derived small RNAs is indicated by different colors. Genomic coverage depth of 21-to 23-nt vsiRNAs is indicated by the position of its 5’ terminal nucleotide. Sense strand-vsiRNAs are depicted in red, and antisense strand-vsiRNAs are presented in blue.
(TIF)

S3 Fig The alignment of *H*. *longicornis* Dicer-like proteins to two Dicer proteins of *I*. *scapularis*. The alignment was performed by Clone Manager with scoring matrix BLOSUM 62. There is 73% identity between *H*. *longicornis* Dicer1-like protein and *I*. *scapularis* Dicer XP_029830052.1 (Dicer90) (A), and 58% identity between *H*. *longicornis* Dicer2-like protein and *I*. *scapularis* Dicer XP_029830051.1 (Dicer89) (B).
(TIF)

S4 Fig Small RNA sequencing of ticks infected with SINV by injection, a repeat of Fig 6B. Size distribution of total reads (left), virus-derived small RNAs (middle) and genomic coverage depth of 21- to 23-nt vsiRNAs (right) sequenced from ticks infected with SINV by injection at 5dpi. Read counts are shown as per million total 18- to 28-nt reads (CPM) and the 5’ terminal nucleotide of virus-derived small RNAs is indicated by different colors. Genomic coverage depth of 21-to 23-nt vsiRNAs is indicated by the position of its 5’ terminal nucleotide. Sense strand-vsiRNAs are depicted in red, and antisense strand-vsiRNAs are presented in blue.
(TIF)

S5 Fig Small RNA sequencing of ticks infected with SINV_NoV B2_ or SINV_NoV mB2_ at 14 dpi by injection, repeats of Fig 6E–6H. (A and B) Size distribution of total reads(left), virus-derived small RNAs(middle) and genomic coverage depth of 21–23 nt vsiRNAs(right) sequenced from ticks after infection with SINV_NoV B2_ (A) and SINV_NoV mB2_ (B). (C) Read counts (CPM) of mature miRNAs and vsiRNAs in the library of SINV_NoV B2_ or SINV_NoV mB2_ infected ticks at 14 dpi. (D) Relative abundance comparison of 21- to 23-nt vsiRNAs sequenced from ticks infected with SINV_NoV B2_ and SINV_NoV mB2_ at 14 dpi. Read counts were normalized either by total 21- to 23-nt reads only (green bar) or by both total 21- to 23-nt reads and viral relative accumulation determined by RT-qPCR (red bar). Read counts are shown as per million total 18- to 28-nt reads (CPM) and the 5’ terminal nucleotide of virus-derived small RNAs is indicated by different colors. Genomic coverage depth of 21-to 23-nt vsiRNAs is indicated by the position of its 5’ terminal nucleotide. Sense strand-vsiRNAs are depicted in red, and antisense strand-vsiRNAs are presented in blue.
(TIF)

S6 Fig The heterologous proteins expression in recombinant SINV infected ticks. Western blotting detection of Flag-tagged NP or NS proteins from ticks infected with SINV_SFTSV NP_ and SINV_SFTSV NS_ by microinjection at 14dpi. Endogenous β-actin as a loading control.
(TIF)

S7 Fig Full-length blots from Figs 3 and 6, S6 Fig. (A and B) Detection of dsRNA and small RNAs by 3% agarose gel with GelRed staining. (C-E) Western blotting detection of input and immune-precipitated Flag-tagged DCL1, DCL2(C), Flag-tagged EGFP (D) ectopically expressing in S2 cells or NoDice 293T cells and endogenous Actin (E) of respective cells. Molecular weight standards are shown on the left. (F and G) Northern blotting detection of rSINV derived vsiRNA (F) and endogenous U6 (G) from ticks infected with SINV_NoV B2_ and SINV_NoV mB2_. (H and I) Western blotting detection of Flag-tagged NP, NS (H) and endogenous Actin (I) from ticks mock or infected with SINV_SFTSV NP_ and SINV_SFTSV NS_. Molecular weight standards are shown on the right. Each experiment was repeated twice with reproducible results.
(TIF)

S1 Table Differential expression analysis between CT 2d, CT 6d, and SFTSV 6d. (XLSX)

S2 Table Alignment of HlDCL-1 and HlDCL-2 with two available *H*. *longicornis* genome databases. (DOCX)

S3 Table Primers related to experimental procedures. (DOCX)

## References

[1] Gulia-Nuss M, Nuss AB, Meyer JM, Sonenshine DE, Roe RM, Waterhouse RM, et al. Genomic insights into the Ixodes scapularis tick vector of Lyme disease. Nat Commun. 2016;7:10507. Epub 2016/02/10. doi: 10.1038/ncomms10507 26856261 PMC4748124

[2] Moyer MW. The growing global battle against blood-sucking ticks. Nature. 2015;524(7566):406–8. doi: 10.1038/524406a 26310749

[3] Jia N, Wang J, Shi W, Du L, Sun Y, Zhan W, et al. Large-Scale Comparative Analyses of Tick Genomes Elucidate Their Genetic Diversity and Vector Capacities. Cell. 2020;182(5):1328–40.e13. doi: 10.1016/j.cell.2020.07.023 . 32814014

[4] de la Fuente J, Villar M, Cabezas-Cruz A, Estrada-Pena A, Ayllon N, Alberdi P. Tick–host–pathogen interactions: conflict and cooperation. Plos pathogens. 2016;12(4):e1005488. doi: 10.1371/journal.ppat.1005488 27099928 PMC4839629

[5] Zhao L, Li J, Cui X, Jia N, Wei J, Xia L, et al. Distribution of Haemaphysalis longicornis and associated pathogens: analysis of pooled data from a China field survey and global published data. The Lancet Planetary health. 2020;4(8):e320–e9. doi: 10.1016/S2542-5196(20)30145-5 . 32800150

[6] Talactac MR, Yoshii K, Hernandez EP, Kusakisako K, Galays RL, Fujisaki K, et al. Vector competence of Haemaphysalis longicornis ticks for a Japanese isolate of the Thogoto virus. Scientific Reports. 2018;8. doi: 10.1038/s41598-017-18329-3 29915199 PMC6006283

[7] Zhang L, Li S, Huang S-J, Wang Z-D, Wei F, Feng X-M, et al. Isolation and genomic characterization of lymphocytic choriomeningitis virus in ticks from northeastern China. Transboundary and Emerging Diseases. 2018;65(6):1733–9. doi: 10.1111/tbed.12946 29992783

[8] Gong S, He B, Wang Z, Shang L, Wei F, Liu Q, et al. Nairobi Sheep Disease Virus RNA in Ixodid Ticks, China, 2013. Emerging Infectious Diseases. 2015;21(4):718–20. doi: 10.3201/eid2104.141602 25811222 PMC4378503

[9] Ko S, Kang J-G, Kim SY, Kim H-C, Klein TA, Chong S-T, et al. Prevalence of tick-borne encephalitis virus in ticks from southern Korea. Journal of Veterinary Science. 2010;11(3):197–203. doi: 10.4142/jvs.2010.11.3.197 20706026 PMC2924480

[10] McMullan LK, Folk SM, Kelly AJ, MacNeil A, Goldsmith CS, Metcalfe MG, et al. A New Phlebovirus Associated with Severe Febrile Illness in Missouri. New England Journal of Medicine. 2012;367(9):834–41. doi: 10.1056/NEJMoa1203378 22931317

[11] Yu X-J, Liang M-F, Zhang S-Y, Liu Y, Li J-D, Sun Y-L, et al. Fever with Thrombocytopenia Associated with a Novel Bunyavirus in China. New England Journal of Medicine. 2011;364(16):1523–32. doi: 10.1056/NEJMoa1010095 21410387 PMC3113718

[12] Li H, Lu Q-B, Xing B, Zhang S-F, Liu K, Du J, et al. Epidemiological and clinical features of laboratory-diagnosed severe fever with thrombocytopenia syndrome in China, 2011–17: a prospective observational study. Lancet Infectious Diseases. 2018;18(10):1127–37. doi: 10.1016/S1473-3099(18)30293-7 30054190

[13] Zhan J, Wang Q, Cheng J, Hu B, Li J, Zhan F, et al. Current status of severe fever with thrombocytopenia syndrome in China. Virologica Sinica. 2017;32(1):51–62. doi: 10.1007/s12250-016-3931-1 28251515 PMC6598917

[14] Kim K-H, Yi J, Kim G, Choi SJ, Jun KI, Kim N-H, et al. Severe Fever with Thrombocytopenia Syndrome, South Korea, 2012. Emerging Infectious Diseases. 2013;19(11):1892–4. doi: 10.3201/eid1911.130792 24206586 PMC3837670

[15] Takahashi T, Maeda K, Suzuki T, Ishido A, Shigeoka T, Tominaga T, et al. The First Identification and Retrospective Study of Severe Fever With Thrombocytopenia Syndrome in Japan. Journal of Infectious Diseases. 2014;209(6):816–27. doi: 10.1093/infdis/jit603 24231186 PMC7107388

[16] Tran XC, Yun Y, Le Van A, Kim S-H, Thao NTP, Man PKC, et al. Endemic Severe Fever with Thrombocytopenia Syndrome, Vietnam. Emerging Infectious Diseases. 2019;25(5):1029–31. doi: 10.3201/eid2505.181463 31002059 PMC6478219

[17] Lu Q-B, Li H, Jiang F-C, Mao L-L, Liu X-S, Wang N, et al. The Differential Characteristics Between Severe Fever With Thrombocytopenia Syndrome and Hemorrhagic Fever With Renal Syndrome in the Endemic Regions. Open Forum Infectious Diseases. 2019;6(12). doi: 10.1093/ofid/ofz477 32128325 PMC7047964

[18] Yu K-M, Park S-J, Yu M-A, Kim Y-I, Choi Y, Jung JU, et al. Cross-genotype protection of live-attenuated vaccine candidate for severe fever with thrombocytopenia syndrome virus in a ferret model. Proceedings of the National Academy of Sciences. 2019;116(52):26900–8. 10.1073/pnas.1914704116 PMC6936527 31818942

[19] WHO. List of Blueprint priority diseases 2018. https://www.who.int/blueprint/priority-diseases/en/xx.

[20] Abudurexiti A, Adkins S, Alioto D, Alkhovsky SV, Avsic-Zupanc T, Ballinger MJ, et al. Taxonomy of the order Bunyavirales: update 2019. Archives of Virology. 2019;164(7):1949–65. doi: 10.1007/s00705-019-04253-6 31065850 PMC6641860

[21] Lei XY, Liu MM, Yu XJ. Severe fever with thrombocytopenia syndrome and its pathogen SFTSV. Microbes Infect. 2015;17(2):149–54. Epub 2014/12/17. doi: 10.1016/j.micinf.2014.12.002 . 25498868

[22] Wu Y, Zhu Y, Gao F, Jiao Y, Oladejo BO, Chai Y, et al. Structures of phlebovirus glycoprotein Gn and identification of a neutralizing antibody epitope. Proceedings of the National Academy of Sciences. 2017;114(36):E7564–E73. doi: 10.1073/pnas.1705176114 28827346 PMC5594662

[23] Luo L-M, Zhao L, Wen H-L, Zhang Z-T, Liu J-W, Fang L-Z, et al. Haemaphysalis longicornis Ticks as Reservoir and Vector of Severe Fever with Thrombocytopenia Syndrome Virus in China. Emerging Infectious Diseases. 2015;21(10):1770–6. doi: 10.3201/eid2110.150126 26402039 PMC4593435

[24] Zhuang L, Sun Y, Cui X-M, Tang F, Hu J-G, Wang L-Y, et al. Transmission of Severe Fever with Thrombocytopenia Syndrome Virus by Haemaphysalis longicornis Ticks, China. Emerging Infectious Diseases. 2018;24(5):868–71. doi: 10.3201/eid2405.151435 29664718 PMC5938789

[25] Park S-W, Song BG, Shin E-H, Yun S-M, Han M-G, Park MY, et al. Prevalence of severe fever with thrombocytopenia syndrome virus in Haemaphysalis longicornis ticks in South Korea. Ticks and tick-borne diseases. 2014;5(6):975–7. doi: 10.1016/j.ttbdis.2014.07.020 25164614

[26] Choi Y, Park S-J, Sun Y, Yoo J-S, Pudupakam RS, Foo S-S, et al. Severe fever with thrombocytopenia syndrome phlebovirus non-structural protein activates TPL2 signalling pathway for viral immunopathogenesis. Nature Microbiology. 2019;4(3):429–37. doi: 10.1038/s41564-018-0329-x 30617349 PMC6548314

[27] Merkling SH, van Rij RP. Beyond RNAi: antiviral defense strategies in Drosophila and mosquito. Journal of insect physiology. 2013;59(2):159–70. doi: 10.1016/j.jinsphys.2012.07.004 22824741

[28] Tikhe CV, Dimopoulos G. Mosquito antiviral immune pathways. Developmental & Comparative Immunology. 2020:103964. doi: 10.1016/j.dci.2020.103964 33301792

[29] Xu J, Cherry S. Viruses and antiviral immunity in Drosophila. Developmental & Comparative Immunology. 2014;42(1):67–84. doi: 10.1016/j.dci.2013.05.002 23680639 PMC3826445

[30] Schneider J, Imler J-L. Sensing and signalling viral infection in Drosophila. Developmental & Comparative Immunology. 2020:103985. doi: 10.1016/j.dci.2020.103985 33358662

[31] Bonning BC, Saleh M-C. The Interplay Between Viruses and RNAi Pathways in Insects. Annual Review of Entomology. 2021;66:61–79. doi: 10.1146/annurev-ento-033020-090410 33417818

[32] Talactac MR, Hernandez EP, Hatta T, Yoshii K, Kusakisako K, Tsuji N, et al. The antiviral immunity of ticks against transmitted viral pathogens. Developmental & Comparative Immunology. 2021:104012. doi: 10.1016/j.dci.2021.104012 33484780

[33] Buchon N, Silverman N, Cherry S. Immunity in Drosophila melanogaster—from microbial recognition to whole-organism physiology. Nature Reviews Immunology. 2014;14(12):796–810. doi: 10.1038/nri3763 25421701 PMC6190593

[34] Li HW, Li WX, Ding SW. Induction and suppression of RNA silencing by an animal virus. Science. 2002;296(5571):1319–21. doi: 10.1126/science.1070948 12016316

[35] Galiana-Arnoux D, Dostert C, Schneemann A, Hoffmann JA, Imler J-L. Essential function in vivo for Dicer-2 in host defense against RNA viruses in drosophila. Nature Immunology. 2006;7(6):590–7. doi: 10.1038/ni1335 16554838

[36] Wang XH, Aliyari R, Li WX, Li HW, Kim K, Carthew R, et al. RNA interference directs innate immunity against viruses in adult Drosophila. Science. 2006;312(5772):452–4. doi: 10.1126/science.1125694 16556799 PMC1509097

[37] Aliyari R, Wu Q, Li H-W, Wang X-H, Li F, Green LD, et al. Mechanism of Induction and Suppression of Antiviral Immunity Directed by Virus-Derived Small RNAs in Drosophila. Cell Host & Microbe. 2008;4(4):387–97. doi: 10.1016/j.chom.2008.09.001 18854242 PMC2584229

[38] Nayak A, Kim DY, Trnka MJ, Kerr CH, Lidsky PV, Stanley DJ, et al. A Viral Protein Restricts Drosophila RNAi Immunity by Regulating Argonaute Activity and Stability. Cell Host Microbe. 2018;24(4):542–57 e9. Epub 2018/10/12. doi: 10.1016/j.chom.2018.09.006 30308158 PMC6450077

[39] Zhang L, Xu W, Gao X, Li W, Qi S, Guo D, et al. lncRNA sensing of a viral suppressor of RNAi activates non-canonical innate immune signaling in Drosophila. Cell Host & Microbe. 2020;27(1):115–28. e8. doi: 10.1016/j.chom.2019.12.006 31917956

[40] Campbell CL, Keene KM, Brackney DE, Olson KE, Blair CD, Wilusz J, et al. Aedes aegypti uses RNA interference in defense against Sindbis virus infection. BMC Microbiol. 2008;8:47. Epub 2008/03/28. doi: 10.1186/1471-2180-8-47 18366655 PMC2278134

[41] Myles KM, Wiley MR, Morazzani EM, Adelman ZN. Alphavirus-derived small RNAs modulate pathogenesis in disease vector mosquitoes. Proceedings of the National Academy of Sciences of the United States of America. 2008;105(50):19938–43. doi: 10.1073/pnas.0803408105 19047642 PMC2604946

[42] Cirimotich CM, Scott JC, Phillips AT, Geiss BJ, Olson KE. Suppression of RNA interference increases alphavirus replication and virus-associated mortality in Aedes aegypti mosquitoes. BMC Microbiol. 2009;9:49. Epub 2009/03/07. doi: 10.1186/1471-2180-9-49 19265532 PMC2660349

[43] Keene KM, Foy BD, Sanchez-Vargas I, Beaty BJ, Blair CD, Olson KE. RNA interference acts as a natural antiviral response to O’nyong-nyong virus (Alphavirus; Togaviridae) infection of Anopheles gambiae. Proceedings of the National Academy of Sciences of the United States of America. 2004;101(49):17240–5. doi: 10.1073/pnas.0406983101 15583140 PMC535383

[44] Li WX, Li HW, Lu R, Li F, Dus M, Atkinson P, et al. Interferon antagonist proteins of influenza and vaccinia viruses are suppressors of RNA silencing. Proceedings of the National Academy of Sciences of the United States of America. 2004;101(5):1350–5. doi: 10.1073/pnas.0308308100 14745017 PMC337056

[45] Ding SW. RNA-based antiviral immunity. Nat Rev Immunol. 2010;10(9):632–44. Epub 2010/08/14. doi: 10.1038/nri2824 . 20706278

[46] Chao JA, Lee JH, Chapados BR, Debler EW, Schneemann A, Williamson JR. Dual modes of RNA-silencing suppression by flock house virus protein B2. Nature Structural & Molecular Biology. 2005;12(11):952–7. doi: 10.1038/nsmb1005 16228003

[47] Nayak A, Berry B, Tassetto M, Kunitomi M, Acevedo A, Deng C, et al. Cricket paralysis virus antagonizes Argonaute 2 to modulate antiviral defense in Drosophila. Nature Structural & Molecular Biology. 2010;17(5):547–U41. doi: 10.1038/nsmb.1810 20400949 PMC3815677

[48] van Cleef KWR, van Mierlo JT, Miesen P, Overheul GJ, Fros JJ, Schuster S, et al. Mosquito and Drosophila entomobirnaviruses suppress dsRNA- and siRNA-induced RNAi. Nucleic Acids Research. 2014;42(13):8732–44. doi: 10.1093/nar/gku528 24939903 PMC4117760

[49] Bronkhorst AW, Vogels R, Overheul GJ, Pennings B, Gausson-Dorey V, Miesen P, et al. A DNA virus-encoded immune antagonist fully masks the potent antiviral activity of RNAi in Drosophila. Proceedings of the National Academy of Sciences. 2019;116(48):24296–302. doi: 10.1073/pnas.1909183116 31712431 PMC6883776

[50] Bronkhorst AW, van Cleef KW, Venselaar H, van Rij RP. A dsRNA-binding protein of a complex invertebrate DNA virus suppresses the Drosophila RNAi response. Nucleic acids research. 2014;42(19):12237–48. doi: 10.1093/nar/gku910 25274730 PMC4231766

[51] Kurscheid S, Lew-Tabor AE, Rodriguez Valle M, Bruyeres AG, Doogan VJ, Munderloh UG, et al. Evidence of a tick RNAi pathway by comparative genomics and reverse genetics screen of targets with known loss-of-function phenotypes in Drosophila. BMC Mol Biol. 2009;10:26. Epub 2009/03/28. doi: 10.1186/1471-2199-10-26 19323841 PMC2676286

[52] Schnettler E, Tykalova H, Watson M, Sharma M, Sterken MG, Obbard DJ, et al. Induction and suppression of tick cell antiviral RNAi responses by tick-borne flaviviruses. Nucleic Acids Research. 2014;42(14):9436–46. doi: 10.1093/nar/gku657 25053841 PMC4132761

[53] Grubaugh ND, Ruckert C, Armstrong PM, Bransfield A, Anderson JF, Ebel GD, et al. Transmission bottlenecks and RNAi collectively influence tick-borne flavivirus evolution. Virus Evolution. 2016;2(2). doi: 10.1093/ve/vew033 28058113 PMC5210029

[54] Griffin D. Alphaviruses. Fields Virology: Sixth Edition: Wolters Kluwer Health Adis (ESP); 2013.

[55] Goic B, Vodovar N, Mondotte JA, Monot C, Frangeul L, Blanc H, et al. RNA-mediated interference and reverse transcription control the persistence of RNA viruses in the insect model Drosophila. Nat Immunol. 2013;14(4):396–403. Epub 2013/02/26. doi: 10.1038/ni.2542 . 23435119

[56] Tassetto M, Kunitomi M, Andino R. Circulating Immune Cells Mediate a Systemic RNAi-Based Adaptive Antiviral Response in Drosophila. Cell. 2017;169(2):314–25 e13. Epub 2017/04/08. doi: 10.1016/j.cell.2017.03.033 28388413 PMC5730277

[57] Samuel GH, Wiley MR, Badawi A, Adelman ZN, Myles KM. Yellow fever virus capsid protein is a potent suppressor of RNA silencing that binds double-stranded RNA. Proceedings of the National Academy of Sciences of the United States of America. 2016;113(48):13863–8. doi: 10.1073/pnas.1600544113 27849599 PMC5137771

[58] Johnson KL, Price BD, Ball LA. Recovery of infectivity from cDNA clones of nodamura virus and identification of small nonstructural proteins. Virology. 2003;305(2):436–51. Epub 2003/02/08. doi: 10.1006/viro.2002.1769 . 12573589

[59] Johnson KL, Price BD, Eckerle LD, Ball LA. Nodamura virus nonstructural protein B2 can enhance viral RNA accumulation in both mammalian and insect cells. Journal of Virology. 2004;78(12):6698–704. doi: 10.1128/JVI.78.12.6698-6704.2004 15163762 PMC416532

[60] Li Y, Lu J, Han Y, Fan X, Ding S-W. RNA Interference Functions as an Antiviral Immunity Mechanism in Mammals. Science. 2013;342(6155):231–4. doi: 10.1126/science.1241911 24115437 PMC3875315

[61] Maillard PV, Ciaudo C, Marchais A, Li Y, Jay F, Ding SW, et al. Antiviral RNA interference in mammalian cells. Science. 2013;342(6155):235–8. Epub 2013/10/12. doi: 10.1126/science.1241930 24115438 PMC3853215

[62] Han Q, Chen G, Wang J, Jee D, Li W-X, Lai EC, et al. Mechanism and Function of Antiviral RNA Interference in Mice. Mbio. 2020;11(4). doi: 10.1128/mBio.03278-19 32753500 PMC7407090

[63] Liu Y, Wu B, Paessler S, Walker DH, Tesh RB, Yu X-j. The pathogenesis of severe fever with thrombocytopenia syndrome virus infection in alpha/beta interferon knockout mice: insights into the pathologic mechanisms of a new viral hemorrhagic fever. Journal of virology. 2014;88(3):1781–6. doi: 10.1128/JVI.02277-13 24257618 PMC3911604

[64] Chen X-P, Cong M-L, Li M-H, Kang Y-J, Feng Y-M, Plyusnin A, et al. Infection and pathogenesis of Huaiyangshan virus (a novel tick-borne bunyavirus) in laboratory rodents. Journal of General Virology. 2012;93:1288–93. doi: 10.1099/vir.0.041053-0 22357748

[65] Jin C, Liang M, Ning J, Gu W, Jiang H, Wu W, et al. Pathogenesis of emerging severe fever with thrombocytopenia syndrome virus in C57/BL6 mouse model. Proceedings of the National Academy of Sciences. 2012;109(25):10053–8. doi: 10.1073/pnas.1120246109 22665769 PMC3382536

[66] Guo Z, Li Y, Ding S-W. Small RNA-based antimicrobial immunity. Nature Reviews Immunology. 2019;19(1):31–44. doi: 10.1038/s41577-018-0071-x 30301972

[67] Lee YS, Nakahara K, Pham JW, Kim K, He ZY, Sontheimer EJ, et al. Distinct roles for Drosophila Dicer-1 and Dicer-2 in the siRNA/miRNA silencing pathways. Cell. 2004;117(1):69–81. doi: 10.1016/s0092-8674(04)00261-2 15066283

[68] Guerrero FD, Bendele KG, Ghaffari N, Guhlin J, Gedye KR, Lawrence KE, et al. The Pacific Biosciences de novo assembled genome dataset from a parthenogenetic New Zealand wild population of the longhorned tick, Haemaphysalis longicornis Neumann, 1901. Data in Brief. 2019;27. doi: 10.1016/j.dib.2019.104602 31656838 PMC6806438

[69] Welker NC, Maity TS, Ye X, Aruscavage PJ, Krauchuk AA, Liu Q, et al. Dicer’s Helicase Domain Discriminates dsRNA Termini to Promote an Altered Reaction Mode. Molecular Cell. 2011;41(5):589–99. doi: 10.1016/j.molcel.2011.02.005 21362554 PMC3061311

[70] Kandasamy SK, Fukunaga R. Phosphate-binding pocket in Dicer-2 PAZ domain for high-fidelity siRNA production. Proceedings of the National Academy of Sciences of the United States of America. 2016;113(49):14031–6. doi: 10.1073/pnas.1612393113 27872309 PMC5150366

[71] Mueller S, Gausson V, Vodovar N, Deddouche S, Troxler L, Perot J, et al. RNAi-mediated immunity provides strong protection against the negative-strand RNA vesicular stomatitis virus in Drosophila. Proc Natl Acad Sci U S A. 2010;107(45):19390–5. Epub 2010/10/28. doi: 10.1073/pnas.1014378107 20978209 PMC2984213

[72] Moriyama M, Igarashi M, Koshiba T, Irie T, Takada A, Ichinohe T. Two Conserved Amino Acids within the NSs of Severe Fever with Thrombocytopenia Syndrome Phlebovirus Are Essential for Anti-interferon Activity. Journal of Virology. 2018;92(19). doi: 10.1128/JVI.00706-18 30021900 PMC6146818

[73] Jiao L, Ouyang S, Liang M, Niu F, Shaw N, Wu W, et al. Structure of Severe Fever with Thrombocytopenia Syndrome Virus Nucleocapsid Protein in Complex with Suramin Reveals Therapeutic Potential. Journal of Virology. 2013;87(12):6829–39. doi: 10.1128/JVI.00672-13 23576501 PMC3676114

[74] Poirier EZ, Goic B, Tome-Poderti L, Frangeul L, Boussier J, Gausson V, et al. Dicer-2-Dependent Generation of Viral DNA from Defective Genomes of RNA Viruses Modulates Antiviral Immunity in Insects. Cell Host Microbe. 2018;23(3):353–65 e8. Epub 2018/03/06. doi: 10.1016/j.chom.2018.02.001 29503180 PMC5857290

[75] Mondotte JA, Saleh M-C. Antiviral immune response and the route of infection in Drosophila melanogaster. Advances in virus research. 2018;100:247–78. doi: 10.1016/bs.aivir.2017.10.006 29551139

[76] Mondotte JA, Gausson V, Frangeul L, Blanc H, Lambrechts L, Saleh M-C. Immune priming and clearance of orally acquired RNA viruses in Drosophila. Nature microbiology. 2018;3(12):1394–403. doi: 10.1038/s41564-018-0265-9 30374170

[77] Smith AA, Navasa N, Yang X, Wilder CN, Buyuktanir O, Marques A, et al. Cross-Species Interferon Signaling Boosts Microbicidal Activity within the Tick Vector. Cell Host & Microbe. 2016;20(1):91–8. doi: 10.1016/j.chom.2016.06.001 27374407 PMC4945435

[78] Xu J, Hopkins K, Sabin L, Yasunaga A, Subramanian H, Lamborn I, et al. ERK signaling couples nutrient status to antiviral defense in the insect gut. Proc Natl Acad Sci U S A. 2013;110(37):15025–30. Epub 2013/08/28. doi: 10.1073/pnas.1303193110 23980175 PMC3773808

[79] Pakpour N, Akman-Anderson L, Vodovotz Y, Luckhart S. The effects of ingested mammalian blood factors on vector arthropod immunity and physiology. Microbes and Infection. 2013;15(3):243–54. doi: 10.1016/j.micinf.2013.01.003 23370408 PMC3602389

[80] Zhu Y, Tong L, Nie K, Wiwatanaratanabutr I, Sun P, Li Q, et al. Host serum iron modulates dengue virus acquisition by mosquitoes. Nature Microbiology. 2019;4(12):2405–15. doi: 10.1038/s41564-019-0555-x 31527795

[81] Nguyet Minh N, Duong Thi Hue K, Trung Vu T, Nguyen Than Ha Q, Tran CNB, Long Vo T, et al. Host and viral features of human dengue cases shape the population of infected and infectious Aedes aegypti mosquitoes. Proceedings of the National Academy of Sciences of the United States of America. 2013;110(22):9072–7. doi: 10.1073/pnas.1303395110 23674683 PMC3670336

[82] Wagar ZL, Tree MO, Mpoy MC, Conway MJ. Low density lipopolyprotein inhibits flavivirus acquisition in Aedes aegypti. Insect Molecular Biology. 2017;26(6):734–42. doi: 10.1111/imb.12334 28718976

[83] Gammon DB, Mello CC. RNA interference-mediated antiviral defense in insects. Current opinion in insect science. 2015;8:111–20. doi: 10.1016/j.cois.2015.01.006 26034705 PMC4448697

[84] Lambrechts L, Saleh M-C. Manipulating Mosquito Tolerance for Arbovirus Control. Cell Host & Microbe. 2019;26(3):309–13. doi: 10.1016/j.chom.2019.08.005 31513769

[85] Sullivan CS, Ganem D. A virus-encoded inhibitor that blocks RNA interference in mammalian cells. J Virol. 2005;79(12):7371–9. Epub 2005/05/28. doi: 10.1128/JVI.79.12.7371-7379.2005 15919892 PMC1143619

[86] Takeda A, Sugiyama K, Nagano H, Mori M, Kaido M, Mise K, et al. Identification of a novel RNA silencing suppressor, NSs protein of Tomato spotted wilt virus. Febs Letters. 2002;532(1–2):75–9. doi: 10.1016/s0014-5793(02)03632-3 12459466

[87] Soldan SS, Plassmeyer ML, Matukonis MK, Gonzalez-Scarano F. La Crosse virus nonstructural protein NSs counteracts the effects of short interfering RNA. Journal of Virology. 2005;79(1):234–44. doi: 10.1128/JVI.79.1.234-244.2005 15596819 PMC538693

[88] Blakqori G, Delhaye S, Habjan M, Blair CD, Sánchez-Vargas I, Olson KE, et al. La Crosse bunyavirus nonstructural protein NSs serves to suppress the type I interferon system of mammalian hosts. Journal of virology. 2007;81(10):4991–9. doi: 10.1128/JVI.01933-06 17344298 PMC1900204

[89] Hemmes H, Lakatos L, Goldbach R, Burgyan J, Prins M. The NS3 protein of Rice hoja blanca tenuivirus suppresses RNA silencing in plant and insect hosts by efficiently binding both siRNAs and miRNAs. Rna-a Publication of the Rna Society. 2007;13(7):1079–89. doi: 10.1261/rna.444007 17513697 PMC1894927

[90] Pierro DJ, Myles KM, Foy BD, Beaty BJ, Olson KE. Development of an orally infectious Sindbis virus transducing system that efficiently disseminates and expresses green fluorescent protein in Aedes aegypti. Insect Molecular Biology. 2003;12(2):107–16. doi: 10.1046/j.1365-2583.2003.00392.x 12653932

[91] Zhang Y, Li Z, Ye Z, Xu Y, Wang B, Wang C, et al. The activation of antiviral RNA interference not only exists in neural progenitor cells but also in somatic cells in mammals. Emerg Microbes Infect. 2020:1–34. Epub 2020/06/25. doi: 10.1080/22221751.2020.1787798 . 32576094 PMC7473182

[92] Li Y, Basavappa M, Lu J, Dong S, Cronkite DA, Prior JT, et al. Induction and suppression of antiviral RNA interference by influenza A virus in mammalian cells. Nat Microbiol. 2016;2:16250. Epub 2016/12/06. doi: 10.1038/nmicrobiol.2016.250 27918527 PMC5488270

[93] Grabherr MG, Haas BJ, Yassour M, Levin JZ, Thompson DA, Amit I, et al. Full-length transcriptome assembly from RNA-Seq data without a reference genome. Nature Biotechnology. 2011;29(7):644–U130. doi: 10.1038/nbt.1883 21572440 PMC3571712

[94] Anders S, Huber W. Differential expression analysis for sequence count data. Genome Biology. 2010;11(10). doi: 10.1186/gb-2010-11-10-r106 20979621 PMC3218662

[95] Ashburner M, Ball CA, Blake JA, Botstein D, Butler H, Cherry JM, et al. Gene Ontology: tool for the unification of biology. Nature Genetics. 2000;25(1):25–9. doi: 10.1038/75556 10802651 PMC3037419

