# Supplementary figures and images for: Antiviral RNA interference in disease vector (Asian longhorned) ticks
Source: PLoS Pathog. 2021 Dec 3;17(12):e1010119. doi: 10.1371/journal.ppat.1010119 (PMC8673602; doi:10.1371/journal.ppat.1010119)

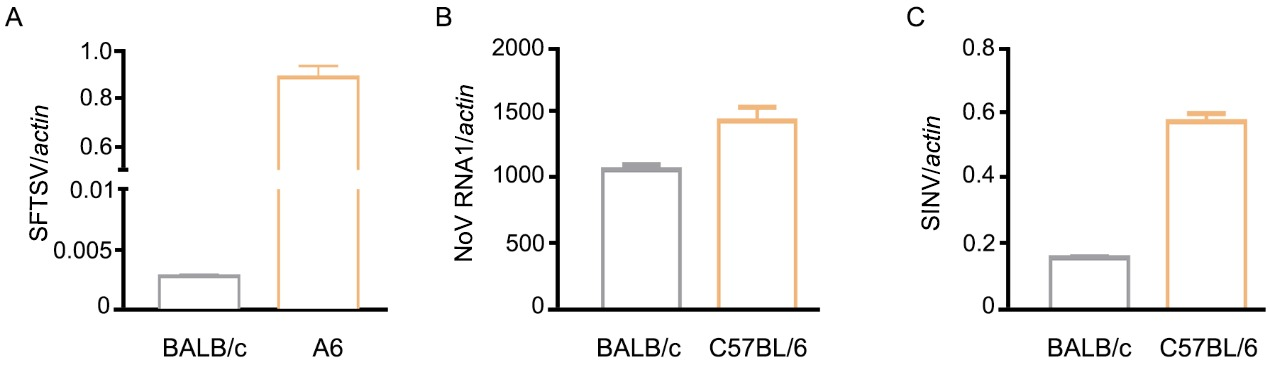

Supplement: S1 Fig — (A) The replication levels of SFTSV in the hind limb of A6 and BALB/c suckling mice at 3 dpi were determined by RT-qPCR. (B) The replication levels of NoV in the hind limb of BALB/c and C57BL/6 suckling mice at 3 dpi were determined by RT-qPCR. (C) The replication levels of SINV in the hind limb of BALB/c and C57BL/6 suckling mice at 3 dpi were determined by RT-qPCR. Total RNA was extracted from the hind limb muscle tissue of mice using TRIzol reagent. The viral replication level was calculated by ΔCt method. β-actin mRNA as the internal reference. (TIF) [file ppat.1010119.s001.tif]

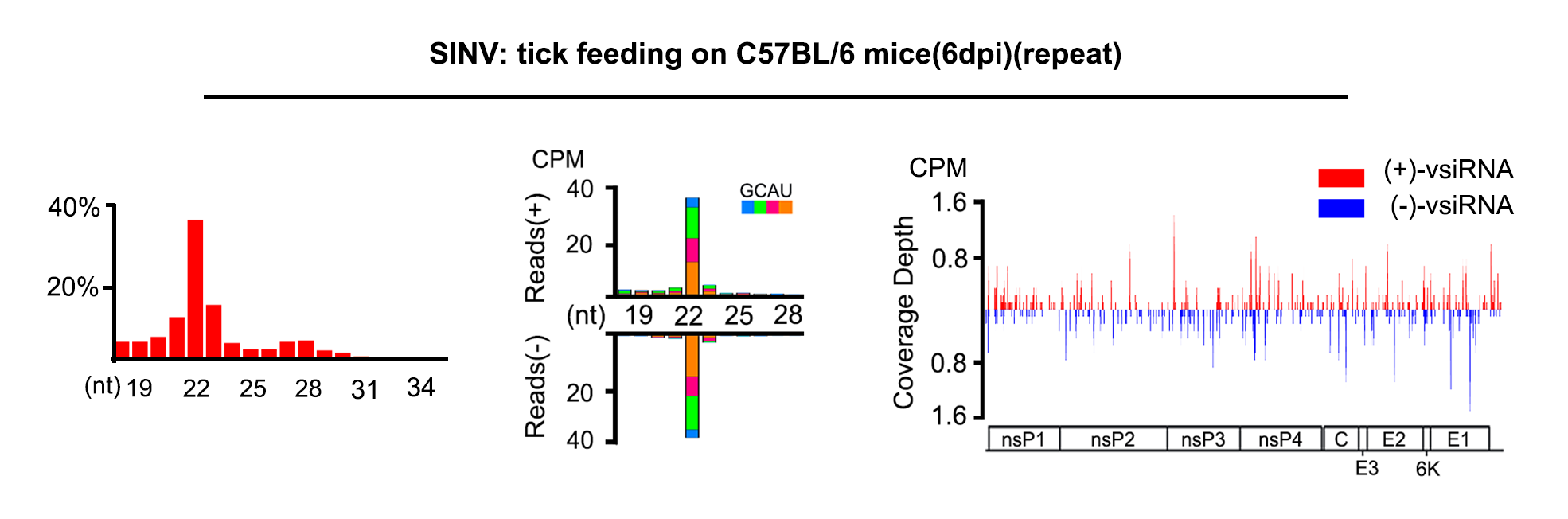

Supplement: S2 Fig — Size distribution of total reads (left), virus-derived small RNAs (middle) and genomic coverage depth of 21- to 23-nt vsiRNAs (right) sequenced from ticks infected with SINV by feeding on C57BL/6 mice at 6dpi. Read counts are shown as per million total 18- to 28-nt reads (CPM) and the 5’ terminal nucleotide of virus-derived small RNAs is indicated by different colors. Genomic coverage depth of 21-to 23-nt vsiRNAs is indicated by the position of its 5’ terminal nucleotide. Sense strand-vsiRNAs are depicted in red, and antisense strand-vsiRNAs are presented in blue. (TIF) [file ppat.1010119.s002.tif]

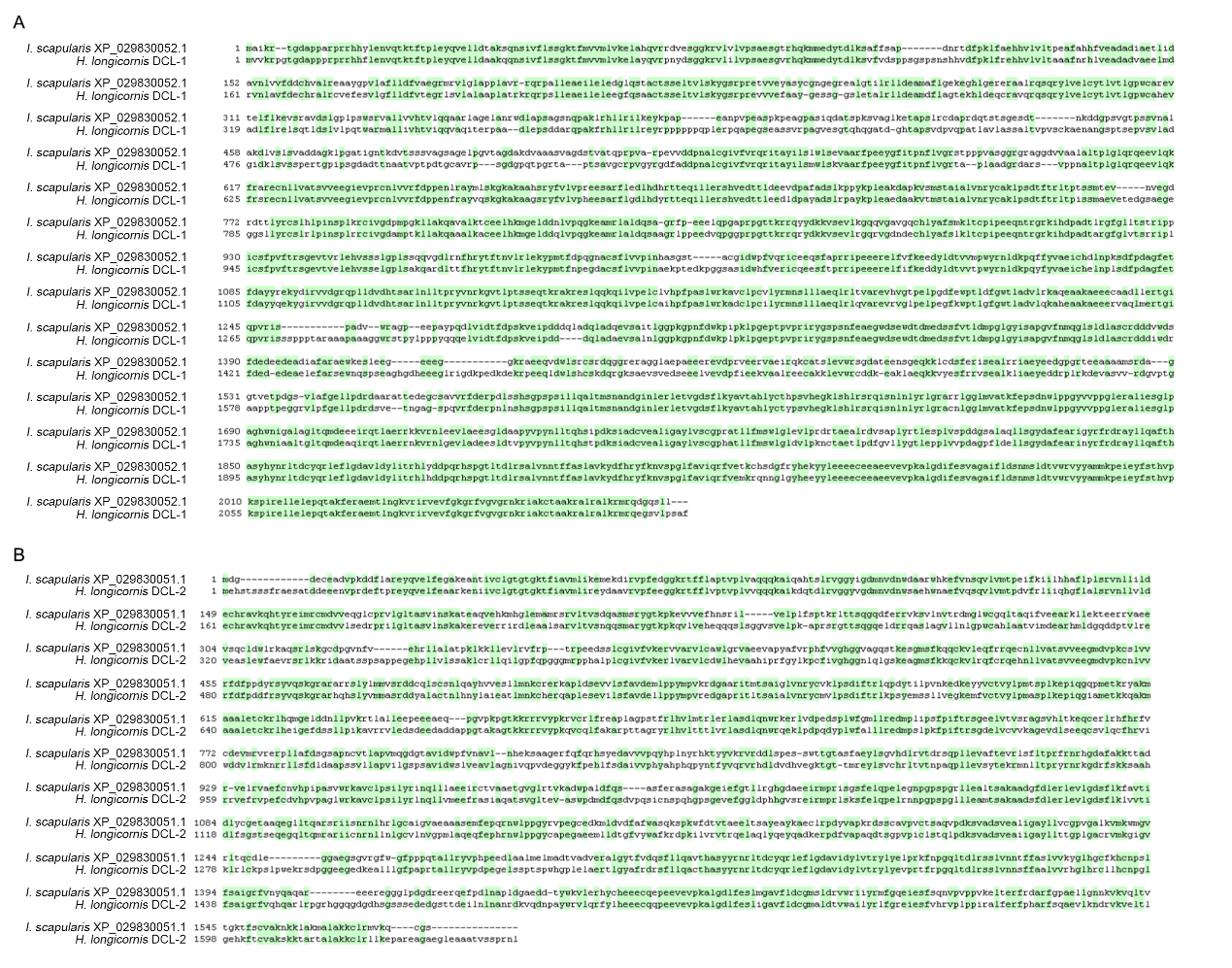

Supplement: S3 Fig — The alignment was performed by Clone Manager with scoring matrix BLOSUM 62. There is 73% identity between H. longicornis Dicer1-like protein and I. scapularis Dicer XP_029830052.1 (Dicer90) (A), and 58% identity between H. longicornis Dicer2-like protein and I. scapularis Dicer XP_029830051.1 (Dicer89) (B). (TIF) [file ppat.1010119.s003.tif]

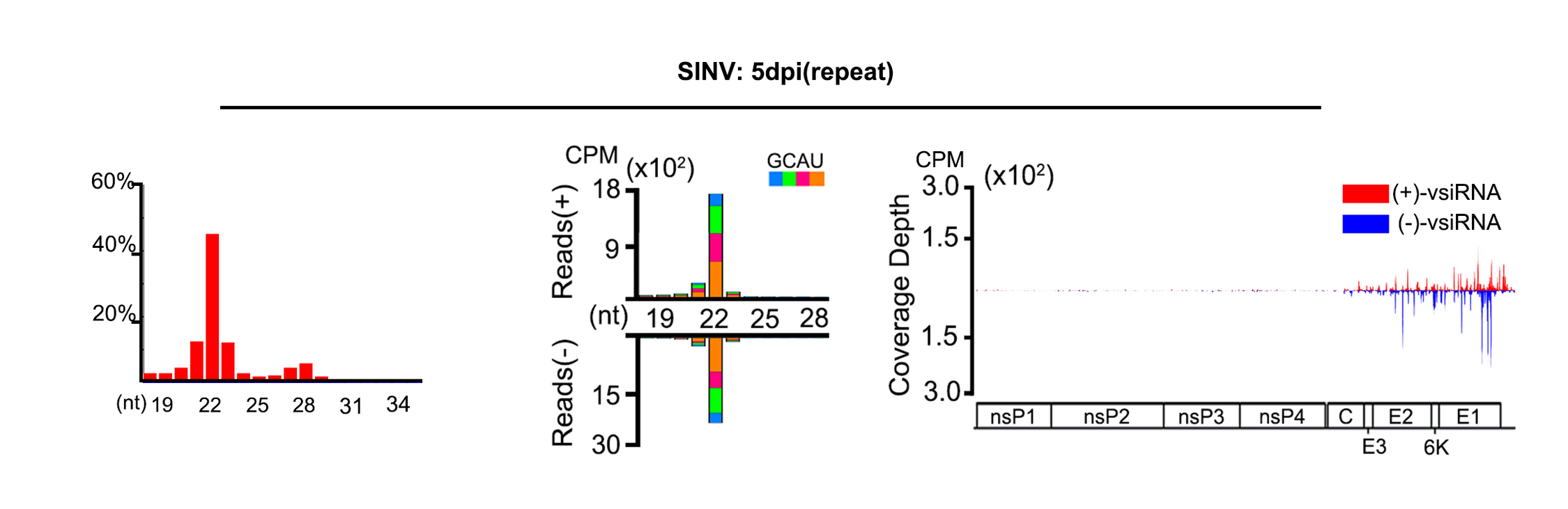

Supplement: S4 Fig — Size distribution of total reads (left), virus-derived small RNAs (middle) and genomic coverage depth of 21- to 23-nt vsiRNAs (right) sequenced from ticks infected with SINV by injection at 5dpi. Read counts are shown as per million total 18- to 28-nt reads (CPM) and the 5’ terminal nucleotide of virus-derived small RNAs is indicated by different colors. Genomic coverage depth of 21-to 23-nt vsiRNAs is indicated by the position of its 5’ terminal nucleotide. Sense strand-vsiRNAs are depicted in red, and antisense strand-vsiRNAs are presented in blue. (TIF) [file ppat.1010119.s004.tif]

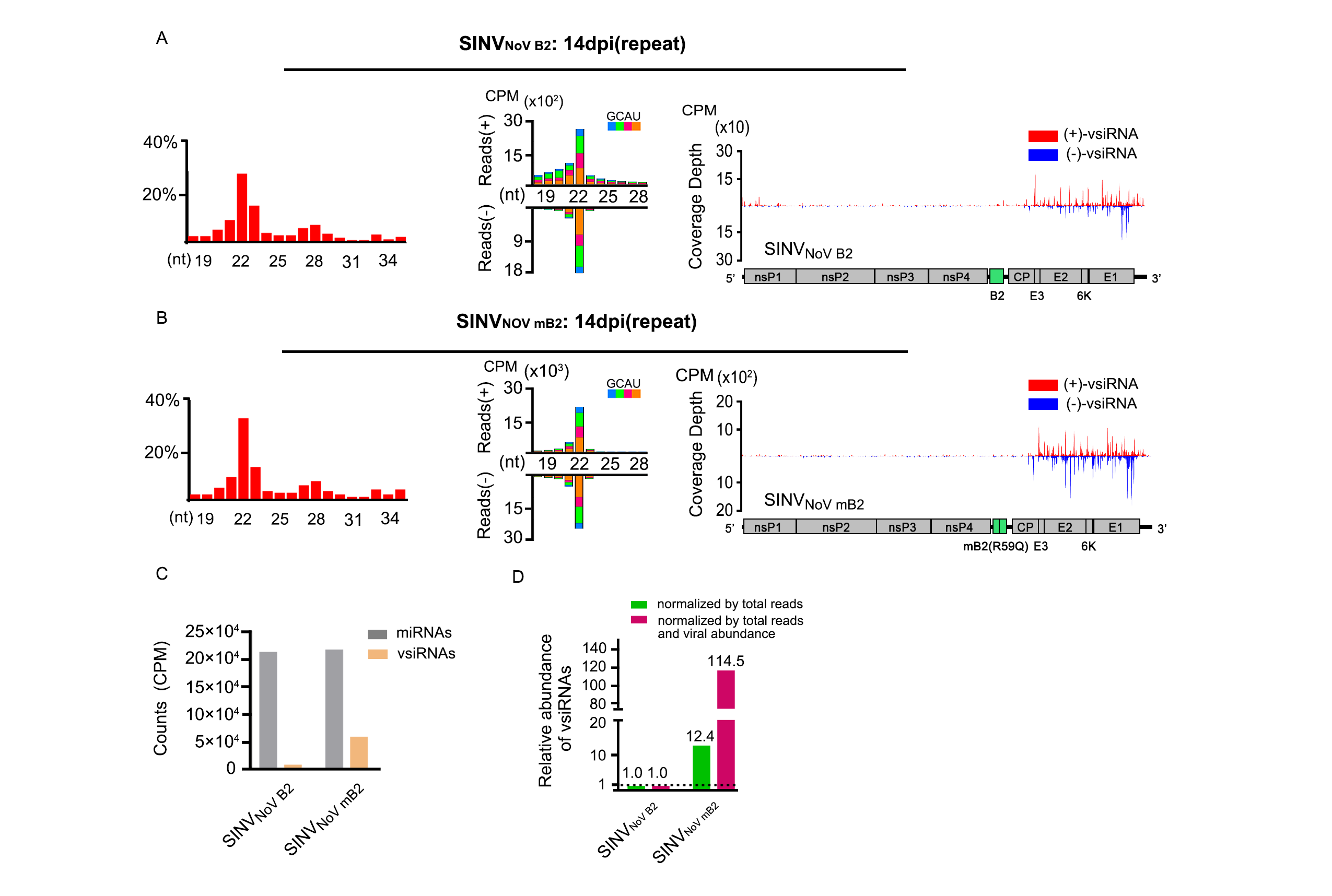

Supplement: S5 Fig — (A and B) Size distribution of total reads(left), virus-derived small RNAs(middle) and genomic coverage depth of 21–23 nt vsiRNAs(right) sequenced from ticks after infection with SINVNoV B2 (A) and SINVNoV mB2 (B). (C) Read counts (CPM) of mature miRNAs and vsiRNAs in the library of SINVNoV B2 or SINVNoV mB2 infected ticks at 14 dpi. (D) Relative abundance comparison of 21- to 23-nt vsiRNAs sequenced from ticks infected with SINVNoV B2 and SINVNoV mB2 at 14 dpi. Read counts were normalized either by total 21- to 23-nt reads only (green bar) or by both total 21- to 23-nt reads and viral relative accumulation determined by RT-qPCR (red bar). Read counts are shown as per million total 18- to 28-nt reads (CPM) and the 5’ terminal nucleotide of virus-derived small RNAs is indicated by different colors. Genomic coverage depth of 21-to 23-nt vsiRNAs is indicated by the position of its 5’ terminal nucleotide. Sense strand-vsiRNAs are depicted in red, and antisense strand-vsiRNAs are presented in blue. (TIF) [file ppat.1010119.s005.tif]

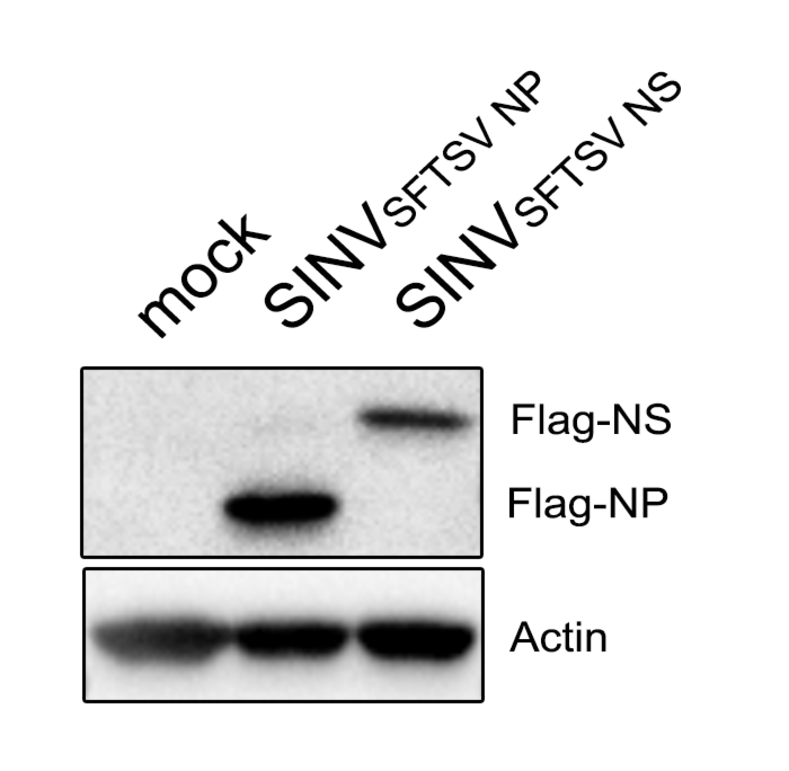

Supplement: S6 Fig — Western blotting detection of Flag-tagged NP or NS proteins from ticks infected with SINVSFTSV NP and SINVSFTSV NS by microinjection at 14dpi. Endogenous β-actin as a loading control. (TIF) [file ppat.1010119.s006.tif]

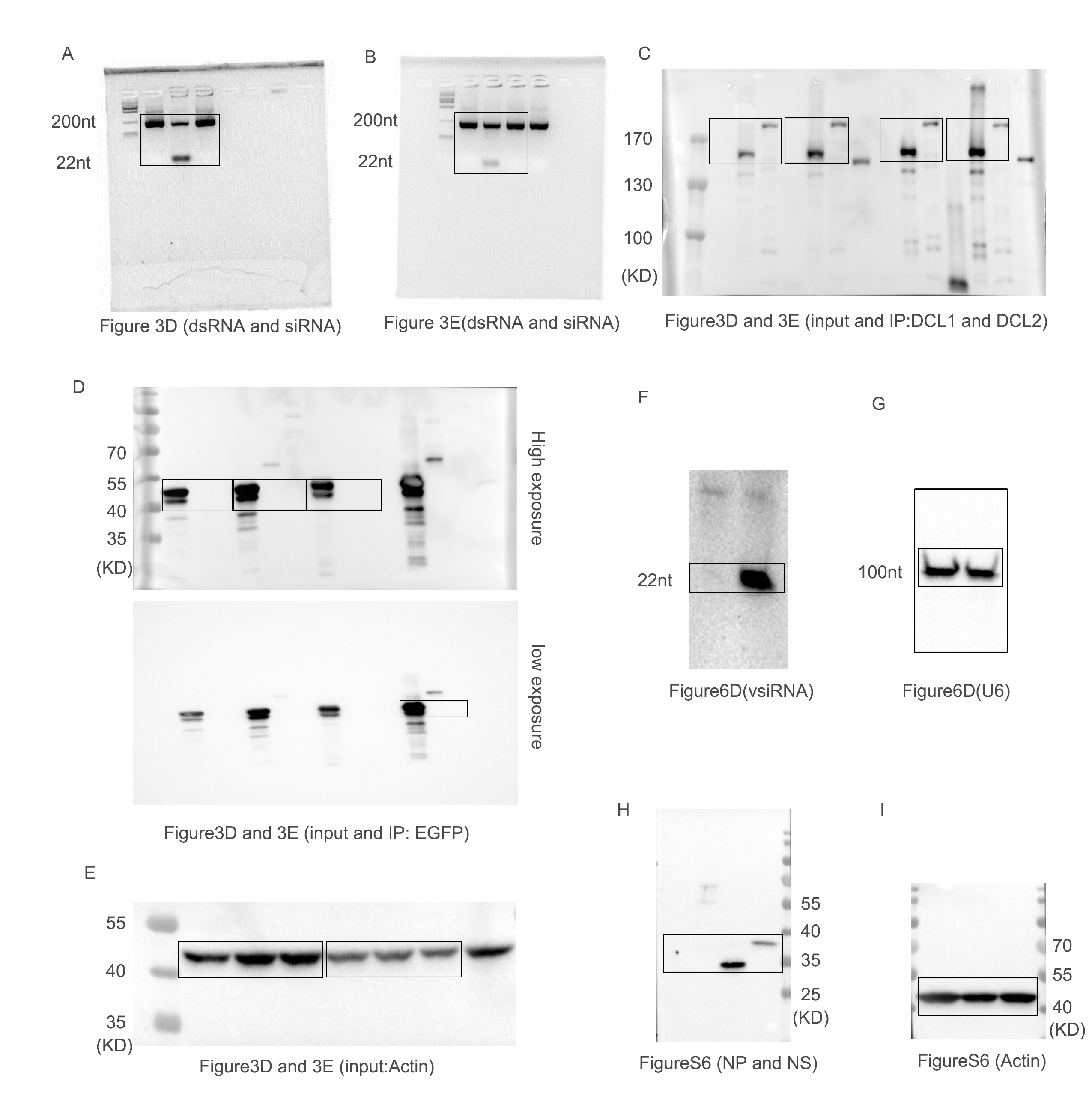

Supplement: S7 Fig — (A and B) Detection of dsRNA and small RNAs by 3% agarose gel with GelRed staining. (C-E) Western blotting detection of input and immune-precipitated Flag-tagged DCL1, DCL2(C), Flag-tagged EGFP (D) ectopically expressing in S2 cells or NoDice 293T cells and endogenous Actin (E) of respective cells. Molecular weight standards are shown on the left. (F and G) Northern blotting detection of rSINV derived vsiRNA (F) and endogenous U6 (G) from ticks infected with SINVNoV B2 and SINVNoV mB2. (H and I) Western blotting detection of Flag-tagged NP, NS (H) and endogenous Actin (I) from ticks mock or infected with SINVSFTSV NP and SINVSFTSV NS. Molecular weight standards are shown on the right. Each experiment was repeated twice with reproducible results. (TIF) [file ppat.1010119.s007.tif]
