# Supplementary material for: Antiviral RNA interference in disease vector (Asian longhorned) ticks
Source: PLoS Pathog. 2021 Dec 3;17(12):e1010119. doi: 10.1371/journal.ppat.1010119 (PMC8673602; doi:10.1371/journal.ppat.1010119)
Supplement: S2 Table — (DOCX) [file ppat.1010119.s009.docx]

S2 Table: Alignment of HlDCL-1 and HlDCL-2 with two available *H. longicornis* genome databases

| CDs | Accession | Query  cover | Chromosome | Max Score | Total  Score | E  Value | Identity% |
| --- | --- | --- | --- | --- | --- | --- | --- |
| DCL-1（6393bp） | JABSTR010000007.1 | 100% | 5 | 3679 | 18011 | 0.0 | 99.28 |
|  | VFIB01000205.1 | 99% | / | 3693 | 11155 | 0.0 | 99.38 |
|  | VFIB01022648.1 | 100% | / | 3626 | 11200 | 0.0 | 98.70 |
|  | VFIB01022713.1 | 100% | / | 3608 | 11040 | 0.0 | 98.46 |
|  |  |  |  |  |  |  |  |
| DCL-2 (4947bp) | JABSTR010000007.1 | 100% | 5 | 1934 | 16238 | 0.0 | 97.23 |
|  | VFIB01006168.1 | 100% | / | 1948 | 8486 | 0.0 | 97.49 |
|  | VFIB01007685.1 | 100% | / | 1930 | 8569 | 0.0 | 97.23 |
|  | VFIB01008134.1 | 100% | / | 1907 | 8446 | 0.0 | 96.71 |
