# Supplementary material for: Antiviral RNA interference in disease vector (Asian longhorned) ticks
Source: PLoS Pathog. 2021 Dec 3;17(12):e1010119. doi: 10.1371/journal.ppat.1010119 (PMC8673602; doi:10.1371/journal.ppat.1010119)
Supplement: S3 Table — (DOCX) [file ppat.1010119.s010.docx]

S2 Table: Primers related to experimental procedures

Primers for PCR

| Name | Sequence(5’ to 3’) |
| --- | --- |
| Forward primer for full-length HlDCL-1 | ATGGTTGTGAAAAGACCTGGGACCGG |
| Reverse primer for full-length HlDCL-1 | CGCAGTAGAAGGCATTTCCACAACACG |
| Forward primer for full-length HlDCL-2 | CAGCCAGCATTCTGCCAAAAGGTGAGC |
| Reverse primer for full-length HlDCL-2 | CCCATCACTACCGACGCCTCGCTACAG |
| Forward primer for HlDCL-2 dsRNA | taatacgactcactatagggGCAGCACCACGGACGAGAT |
| Reverse primer for HlDCL-2 dsRNA | taatacgactcactatagggTGAGCAAGCGGAGGCACTTC |
| Forward primer for GFP dsRNA | taatacgactcactatagggTCAGTGGAGAGGGTGAAG |
| Reverse primer for GFP dsRNA | taatacgactcactatagggCTAGTTGAACGGATCCATC |
| Forward primer for Ago2-like dsRNA | taatacgactcactatagggTGAGCGAGTCTATGACCACCAACA |
| Reverse primer for Ago2-like dsRNA | taatacgactcactatagggACTCATTGTGGTGCTGCTGGG |

Primers for qRT-PCR

| Name | Sequence(5’ to 3’) |
| --- | --- |
| SFTSV-NP forward | CGGAGCCAGCAAGACAGAAGTT |
| SFTSV-NP reverse | TAAGCAGCAGCAGCAACCTCAG |
| SINV nsP2 forward | CTTGTGGTGAAGTCCTACGGTTACG |
| SINV nsP2 reverse | GTGTGCGGCTGTTGTCTAGTTGT |
| NoV RNA1 forward | ccgttcatggcttacacctt |
| NoV RNA1 reverse | gcaccagtcccaaacttcat |
| Tick actin forward | GCCGCCTCCTCCTCTTCC |
| Tick actin reverse | GATGCCGCACGATTCCATACC |
| Mice actin forward | attggcaacgagcggttcc |
| Mice actin reverse | agcactgtgttggcatagagg |
| AGO forward | CATTGTCGAGGGCCAGCCATAC |
| AGO reverse | AGTCCGCCGGATCATGTTGGT |
| Dicer forward | TCCTGGAGTTGGAGCCTCAGA |
| Dicer reverse | CCTTGCCGTTCAGTGTCATC |
| Spätzle forward | CGACCCGCTGTGCTACCAAGTA |
| Spätzle reverse | GCTCGACACGGTTGTCCTCAGT |
| TLR forward | AATCGAGGCTGTCCAGGTCACA |
| TLR reverse | ATGCTCAGAGGCTGCGTCAATG |
| Myd88 forward | CCACAGCTTCCAGTTGCAGACA |
| Myd88 reverse | AGACCGACACCTCAGACCGATG |
| Tube forward | AGACGGCACGACCAGAACAGA |
| Tube reverse | GTCCATCCGAGGCGAGACAATG |
| Pelle forward | GCTGCCGCATTGACAACATCCT |
| Pelle reverse | AGCCGTTCCGCATGTACTGGTA |
| Dorsal forward | GCGTTCAGTCAGACCGTCAGGA |
| Dorsal reverse | ATGCGTGCTTCTCAACCGACTC |
| Cactus forward | TTGCGGTCATGGAAGGCTGGA |
| Cactus reverse | CCGTCCAAGGTCGTTCTGGATG |
| IKKꞵ forward | GAAACCTGGCAGGGCATGGAAG |
| IKKꞵ reverse | TTGAAGTTGGACACGCCGATGG |
| TAB2 forward | GCGAGAAGTTTGGCGAGTACCG |
| TAB2 reverse | GCCTTGGAGCAGTGGAAGTCCT |
| Relish forward | GCAGCGAGCCCTTTGAGTTCA |
| Relish reverse | CAGTGAGGAGCCAAGACGGTGA |
| Uev1a forward | AGCAATGGTGGTGTGGAGAAGC |
| Uev1a reverse | GCAGCACCGTCTTGATGGAGT |
| IAP2 forward | ACCAGCAGGCGACACAACTTTG |
| IAP2 reverse | CGTTGCAGGCTTCATGCCGTTA |
| Dome forward | CGGACACGGGCAACTACAACTG |
| Dome reverse | TCTACTGGAAGCGGTGGCTCTC |
| JAK forward | ATGCCTTCTTCAGCGTCGATGG |
| JAK reverse | CCTTCCTGGCTTCCCTGGTACA |
| STAT forward | GTCAGGCAGAGGACATGCTTCG |
| STAT reverse | CGTGAAGCCAAGCGATGGTGAC |
| SOCS forward | GCTTCACGCAATGACGCCAAC |
| SOCS reverse | CGTCCGCACCGCTACATTCA |
| HlDCL-1 forward | TCCTGGAGTTGGAGCCTCAGA |
| HlDCL-1 reverse | CCTTGCCGTTCAGTGTCATC |
| HlDCL-2 forward | TGCACGCACGCCTCCTACTA |
| HlDCL-2 reverse | GCGTCACCAGGTAGTCGATCAC |
| AMP-AB105544 forward | TAGACTAGCCCAGGTACTCAGAG |
| AMP-AB105544 reverse | TGCGGAAACTTCAGGATGACTTG |
| AMP-EF432731 forward | ACACGGAACCACTTTATCACACC |
| AMP-EF432731 reverse | GAATGGTATGTAACGGCACCCTC |
| Upd1 forward | GCTAGAGGCGAAACACCTATAACC |
| Upd1 reverse | GGGAGTGACAGTAGAAGTGATTTCTG |
| Upd2 forward | GACATTCACACAGACACGCAAC |
| Upd2 reverse | CTGGCTCATAGGGTGTGTAAAC |
| Upd3 forward | GTTAATGGACTCGGCTGTAACACC |
| Upd3 reverse | CATTCATCTCGCCCCCGAATC |
| Vago forward | TTCCTCGCTGAAACTACGCTGAC |
| Vago reverse | TAACACACTTCCCAGACTCCAGC |
| sting-1 forward | GTAATGGGAAGGAGTCGCATAGTC |
| sting-1 reverse | TGGGCTCCTGTTGAAGGTATGAAC |
| sting-2 forward | ATGAGAAAGGACCCTAAACAGCG |
| sting-2 reverse | CCGAGAAGTTCGTGAGTCATTCC |
